# Opportunities and challenges in integrating family planning and nutrition services in Tanzania: a mixed-methods study

**DOI:** 10.1136/bmjgh-2024-017484

**Published:** 2026-04-13

**Authors:** Yohana Laiser, Sachin Shinde, Innocent Yusufu, Uttara Partap, Iddajovana Kinyonge, Jolly Ibrahim, Yumeng Zhang, Mary Mwanyika Sando, Iqbal Shah, Wafaie Fawzi

**Affiliations:** 1 Africa Academy for Public Health, Dar es Salaam, Tanzania, United Republic of; 2 Global Health and Population, Harvard T H Chan School of Public Health, Boston, Massachusetts, USA; 3 Center for Inquiry into Mental Health, Pune, Maharashtra, India

**Keywords:** Nutrition, Maternal health

## Abstract

**Background:**

Integration of family planning (FP) and nutrition is critical for improving maternal and child health, yet evidence on effective integration remains limited in Tanzania. This study explored challenges and opportunities for integrated service delivery in Tanzania.

**Methods:**

A mixed-methods approach was used, combining analysis of the 2022 Tanzania Demographic and Health Survey (n=15, 254 women), a desk review of 25 national documents, and qualitative data from 18 focus group discussions and 14 key informant interviews in urban and rural areas. Thematic analysis and the Walt and Gilson framework guided qualitative and policy reviews, while Poisson regression assessed quantitative associations.

**Results:**

Unmet FP needs were high (25%). Anaemia affected 40% of women, with 6% underweight and 30% overweight/obese. Our novel quantitative analysis revealed that hormonal contraceptive use was associated with 30%–40% reduction in anaemia risk, while short birth intervals doubled the risk of being underweight. Urban women faced higher obesity rates (24%) than rural women (12%). While national policies conceptually support integration, gaps in implementation, monitoring and cross-sectoral coordination persist, including siloed programme design, fragmented governance and a lack of joint operational guidance and monitoring frameworks. Stakeholders highlighted challenges to service integration, including fragmentation between FP and nutrition services, frequent stockouts, long wait times, stigma and limited male engagement. Key opportunities identified include strong community preference for bundled services, advocacy from faith leaders for mosque-based and church-based outreach, and the potential of digital platforms and private-sector partnerships to expand access.

**Conclusions:**

Addressing integration gaps requires policies that prioritise underserved groups (eg, rural women, adolescents) and delivery models tailored to local contexts (urban vs rural), as well as strengthened implementation. Unified governance and community-based platforms, with integrated service delivery at health facilities and in the community, can enhance impact in Tanzania and similar settings.

**WHAT IS ALREADY KNOWN ON THIS TOPIC**

- Family planning (FP) and nutrition are closely linked in improving maternal and child health. Despite policy support, integration of FP and nutrition services in Tanzania is hindered by high unmet needs, anaemia prevalence and persistent implementation, system and sociocultural challenges. Previous quantitative analyses have primarily described trends in FP and nutrition indicators separately.

**WHAT THIS STUDY ADDS**

- **Novel evidence on FP-nutrition links:** Quantifies associations between specific contraceptive methods (eg, hormonal contraceptives) and nutritional outcomes (anaemia, underweight) using 2022 national data, moving beyond descriptive trend analysis.
- **Policy and implementation gaps:** Identifies systemic barriers to integrating FP and nutrition services, including fragmented governance, stockouts and sociocultural stigma, despite existing policy support for integration.
- **Stakeholder-driven solutions:** Proposes actionable strategies like bundled services, faith-based outreach and digital platforms, emphasising community-centred models to enhance integrated care delivery.

**HOW THIS STUDY MIGHT AFFECT RESEARCH, PRACTICE OR POLICY**

- **Research:** Identifies links between FP use and nutrition outcomes, underscoring the need for longitudinal and cost-effectiveness studies to inform integrated service models.
- **Practice:** Supports bundled FP-nutrition services, provider training and community-based delivery to enhance access and service uptake.
- **Policy:** Highlights implementation and coordination gaps, calling for unified governance, domestic financing and integrated monitoring aligned with national strategies.

## Introduction

Family planning (FP) and nutrition are fundamental determinants of maternal, child and adolescent health—particularly in resource-limited settings where the burden of morbidity and mortality is substantial. A recent study examined trends and factors influencing the unmet need for FP among married Tanzanian women (1999–2016) using Tanzania Demographic and Health Survey (TDHS) data.^1^ The unmet need for birth spacing remained stable, with the highest prevalence among women aged 15–24 (21.3%) and the lowest among those aged 35–49 (3.6%).^1^ The unmet need for limiting births was highest among women with five or more children (18.1%) and lowest among those aged 15–24, declining from 9.5% in 1999 to 6.6% in 2016.^1^ Key factors associated with unmet FP needs included health facility visits, fertility intentions and sociodemographic characteristics.^1^ Women who visited health facilities had higher unmet needs, possibly reflecting unfulfilled demand among those actively seeking services. Unmet needs were also significantly higher among women desiring to limit or space births but lacking access to contraceptives, while rural residence and higher parity increased unmet needs; in contrast, older age, employment, media exposure and decision-making autonomy were associated with reduced odds.

Concurrently, nutritional challenges, especially among adolescents and women, continue to impact health outcomes. As per the Tanzania National Health Survey 2018,^2^ 7.3% of non-pregnant women aged 15–49 years were underweight (body mass index (BMI), <18.5%), with higher prevalence in younger age groups (14.8% for 15–19 years) and specific regions such as Kigomo, Kilimanjaro, Rukwa, Ruvuma, Shinyanga and Singida, exceeding 10%. In contrast, 31.7% of non-pregnant women of reproductive age (WRA) were overweight or obese, with obesity prevalence rising from 11.3% in 1991–1992 to 31.7% in 2018, peaking in regions like Dar es Salaam (24.0%) and Stone Town (26.0%). Overnutrition increased with age, with obesity prevalence ranging from 1.9% (15–19 years) to 21.0% (45–49 years). Nationally, anaemia prevalence among WRA decreased from 44.8% in 2015–2016 to 28.8% in 2018, driven by reduced mild anaemia, with the highest prevalence in Pemba South (49.2%) and Mwanza (38.4%) and the lowest in Kilimanjaro (13.5%) and Iringa (16.8%).^2^ These statistics highlight the pressing need to improve FP and nutrition services among WRA in Tanzania.

Recognising the importance of a holistic approach to maternal and child health, there has been growing interest globally in integrating FP and nutrition services with other healthcare interventions.^3–6^ Studies in low-income and middle-income countries (LMICs) have demonstrated the benefits of integrating FP into postpartum care, HIV services and other healthcare programmes.^7–11^ Similarly, nutrition strategies are a key element of maternal and child healthcare services, including antenatal care.^12 13^ Integration can enhance service delivery efficiency, improve health outcomes and optimise resource utilisation. However, despite its potential, the integration of FP and nutrition services faces challenges, particularly in sub-Saharan Africa, due to inadequate healthcare provider training, resource shortages and fragmented service delivery systems.^10 14 15^ In Tanzania, where both FP and nutrition concerns are significant, addressing these challenges is crucial for improving maternal and child health outcomes.

Current understanding of the integration of FP and nutrition services in Tanzania remains limited.^16 17^ While policies acknowledge the interconnectedness of FP and nutrition,^18 19^ their implementation in a coordinated manner has been inadequate. Existing evidence on the current burden and interrelation between suboptimal FP and nutrition, and on integrated FP and nutrition services, is scarce, with limited to no research exploring the effectiveness, feasibility and impact of such integration in Tanzania. Understanding the current landscape and gaps in service delivery, policy implementation and healthcare system readiness is essential for designing evidence-based interventions that can effectively address both FP and nutrition challenges in the country.

This study seeks to address critical gaps in the integration of FP and nutrition services in Tanzania by providing a comprehensive, multidimensional country-based analysis. Specifically, the study: (1) investigates the prevalence of FP use, unmet need for FP and various forms of malnutrition among WRA (15–49 years). Our analysis extends previous trend studies by quantitatively examining the specific associations between FP use (eg, method type) and distinct nutritional outcomes (anaemia, underweight, overweight/obesity) using the latest nationally representative data; (2) conducts a desk review of existing policies and programmes to assess the current state of FP-nutrition integration, identifying key opportunities for improvement and strategic enhancement and (3) explores stakeholder perspectives on FP and nutrition services, delving into their experiences and perceptions of the value and impact of integrated services at the community and national levels.

## Methods

### Study design

This study employed a complementary research design, combining quantitative analysis of the 2022 TDHS with qualitative methods. The qualitative component included a desk review of relevant FP and nutrition programme and policy documents, as well as qualitative investigations through focus group discussions (FGDs) and key informant interviews (KIIs) with a range of stakeholders.

### Demographic and health survey analysis

We analysed data from the 2022 TDHS to explore population trends and needs related to FP and nutrition among WRA. The 2022 TDHS dataset was obtained from the publicly available Demographic and Health Surveys (DHS) Program repository (https://dhsprogram.com) on registration and approval of a research proposal outlining the intended use of the data for this analysis. The analysis focused on a sample of 15,254 WRA, examining trends in FP practices, nutritional status and associated health indices. Key indicators included current contraceptive use, unmet need for FP, marital status among adolescents, age at first sex, age at marriage, parity, average birth intervals, anaemia status and BMI. Among sociodemographic factors, education, age, wealth quintile and urban/rural status were also examined.

We used predefined DHS indicators, or constructed variables according to established definitions (online supplemental file 1). Statistical analysis involved cross-tabulations and Poisson regression models accounting for sampling weights, stratification and clustering, to assess the prevalence of anaemia, underweight and overweight/obesity, and examine associations with FP and reproductive health factors. Poisson models adjusted for age, education, wealth and urban/rural status. All analyses were conducted using Stata V.16 and V.18 (StataCorp).

10.1136/bmjgh-2024-017484.supp1 Supplementary data

### Policy and programme review

In line with WHO definitions,^20^ health policies and programmes are crucial for setting strategic priorities and addressing public health needs. In our study, policies are defined as a system of values, principles and objectives aimed at improving health and reducing burdens, with a clear government vision and priority areas. Programmes are interventions designed to promote health, prevent illness, treat problems and support recovery, requiring proper design, budgeting, monitoring and evaluation. Strategic plans and guidelines outline the government’s priorities, goals and methods for the next 3–5 years, including how success will be measured.

A desk review was conducted of FP and nutrition-related policies and programmes implemented in Tanzania between 2006 and 2022. A set of keywords related to FP, contraceptive use, nutrition, anaemia and relevant strategies was used to identify policy and programme documents from the official websites of Government Ministries, including the Ministries of Health, Finance and Planning, Agriculture and Education, Science and Technology. A total of 25 documents were identified and reviewed, including 7 policy documents and guidelines,^21–27^ and 18 programme reports.^28–45^ In reviewing integrated FP and nutrition policies and programmes, the Walt and Gilson policy analysis triangle framework^46^ was employed to examine the context, content, and development and implementation processes of these documents in Tanzania, along with the actors involved in these processes. A standardised data extraction form was developed to capture key information from the documents, with the framework analysis exploring four elements: context (why the policy is needed), content (the policy’s focus), process (how the policy was developed and implemented) and actors (who participates in and influences its formulation and execution).

### Qualitative investigation

To explore the integration of FP and nutrition services in Tanzania, qualitative data were collected from stakeholders in two settings: Ukonga ward in urban Dar es Salaam and Magomeni ward in rural Bagamoyo district. These sites were purposively selected to capture contrasting socio-economic and health service contexts in Tanzania. Ukonga represents a densely populated urban area with greater access to health facilities but challenges such as overcrowding and longer wait times. Magomeni ward of Bagamoyo district reflects typical rural conditions in Tanzania, characterised by lower population density, limited health infrastructure and services, and greater reliance on community-based services. These sites were chosen to provide perspectives across the urban-rural continuum, enhancing the transferability of findings to similar settings in Tanzania.

KIIs and FGDs were conducted to explore stakeholders’ perspectives on existing FP and nutrition policies, programmes and practices, as well as perceived gaps in design, implementation, monitoring and evaluations. Participants were purposively sampled to ensure maximum variation by age, gender and occupation, and included unmarried adolescent girls (15–19 years), married and childbearing adolescent girls (15–19 years), married adolescents who had not yet begun childbearing, married and unmarried WRA, male and female family members and healthcare providers, including nurses and community health workers. FGD participants from Ukonga ward were identified and recruited through the existing Health and Demographic Surveillance System database, which facilitated selection based on desired characteristics such as age, gender, marital status and childbearing status. Participants from Magomeni ward were recruited through local networks, with the research team collaborating with community leaders (village chairpersons and ward executive officers) to identify village members who met the demographic criteria and inviting them to participate in discussions. KII participants were identified based on their institutional expertise and initially contacted via email by the study coordinator. When institutional heads (eg, directors of government departments, non-government organisations (NGOs) or partner agencies) were unavailable, they were asked to nominate a suitable representative from their institution or department.

FGDs and KIIs were conducted in person in private community or institutional settings. FGDs were facilitated by two trained female research assistants and typically lasted 60–90 min, while KIIs were conducted by two senior researchers and lasted 45–75 min. Semistructured topic guides were used, tailored to stakeholder groups but focused on overlapping themes: experiences with and barriers to accessing FP and nutrition services, perceptions of integrated care, and recommendations for effective service delivery. The guides for FGDs and KIIs are provided in online supplemental file 2.

10.1136/bmjgh-2024-017484.supp2 Supplementary data

All FGD and KII facilitators held at least a bachelor’s degree in social sciences or public health and received intensive training on qualitative methods, research ethics and the use of semi-structured guides. Data collection was supervised by senior researchers (YL and IY) who conducted regular debriefing meetings and provided ongoing support, reviewed transcripts for completeness and held regular debriefings to ensure adherence to the study protocol and consistency in data collection. To ensure reliability, all FGDs and KIIs were conducted in Swahili or English, audio-recorded, transcribed verbatim and translated into English as needed.

We employed a rigorous thematic analysis to systematically examine the qualitative data.^47 48^ An initial coding framework was developed deductively, informed by the study objectives and interview guides, and inductively, allowing new concepts to emerge from the data. Codes captured key domains including perceptions of FP, nutrition, food security, policy and programme design and implementation, stakeholder roles and coordination, contextual facilitators and barriers, and perceived gaps and opportunities for integration of FP and nutrition. Through iterative coding and constant comparison, related codes were grouped into higher-order categories and themes that reflected recurring patterns across stakeholder groups and data sources. Data were independently coded by at least two members of the research team with qualitative research expertise, with regular meetings held to discuss discrepancies, refine the coding framework and agree on theme definitions. Interpretations were developed collaboratively through team discussions, supported by analytic memos and illustrative quotations, to ensure credibility, reflexivity and consistency in the analytic process.

### Mixed-methods data integration

This study employed a mixed-methods design with the primary purpose of triangulation, where quantitative and qualitative data were collected and analysed concurrently but independently, and then integrated during the interpretation phase to provide a more comprehensive understanding of the research problem.^49^ Integration was achieved through a joint display of findings, in which TDHS results and policy review evidence were systematically compared with themes from FGDs and KIIs. Quantitative findings on unmet FP need and anaemia were examined alongside qualitative accounts of stockouts, stigma and long wait times, while policy support for integration was contrasted with stakeholder reports of fragmented implementation. This approach enabled identification of convergence, complementarity and a more nuanced evidence base to inform the study’s conclusions and recommendations. The results of this integration, highlighting convergence and divergence between data sources, are presented in a dedicated subsection at the end of the Results.

We are a multidisciplinary team of global health researchers and practitioners with expertise spanning implementation science, public health, nutrition and sexual and reproductive health. Our team includes academic scholars, programme implementers and policy advisors, with most members based in or working primarily in LMICs. This composition brought contextual relevance and grounded insights into LMIC health systems. Our shared commitment to interdisciplinary integration, equity and evidence-informed policy likely shaped the study’s focus on identifying systemic gaps and advocating for integrated solutions. To manage potential bias, we maintained a diverse team (Tanzanian and international, researchers and practitioners), employed structured analytical frameworks (eg, Walt and Gilson triangle), and held regular consensus discussions to challenge interpretations and ensure findings were grounded in the data. Further reflections on our positionalities, biases and roles in shaping this review are provided in online supplemental file 3.

10.1136/bmjgh-2024-017484.supp3 Supplementary data

This study was reported according to the Good Reporting of A Mixed Methods Study and Consolidated Criteria for Reporting Qualitative Research checklists, provided in online supplemental file 4.

10.1136/bmjgh-2024-017484.supp4 Supplementary data

### Patient and public involvement

This study engaged WRA, health workers, government officials and faith leaders in Tanzania through FGDs and KIIs to help shape research priorities and interpret findings. Participants from both urban (Ukonga Ward) and rural (Magomeni Ward) settings shared insights on barriers to integrated FP and nutrition services, grounding the study in lived experiences. Their perspectives directly informed the study’s recommendations and were integrated into the manuscript’s discussion.

## Results

### Quantitative analysis

TDHS 2022 estimates indicated that 69.1% (95% CI 67.9% to 70.3%) of WRA were not using a contraceptive method at the time of the survey. Among contraceptive users, implants were most commonly used (11.1%, 95% CI 10.4% to 11.8%), followed by injections (7.0%, 95% CI 6.4% to 7.6%), periodic abstinence (3.5%, 95% CI 3.1% to 3.9%) and oral contraceptive pills (2.1%, 95% CI 1.8% to 2.4%). About 2% of WRA used traditional methods. Around one-quarter of WRA had an unmet need for FP, and over one-tenth of adolescents were currently married. Age at first sex was less than 20 years for over 80.0% of WRA, and over half of WRA had their first birth by 20 years of age. About 60% of WRA had parity of 2 or higher, and about one-tenth had a birth interval of less than 24 months (table 1). Use of certain contraceptive methods (male condoms and intrauterine devices) increased with education and wealth while unmet need for FP, early age at first sex or birth, higher parity and shorter birth intervals decreased with higher education and wealth (online supplemental tables 1 and 2). All FP and related indicators were notably less optimal in rural than in urban areas (online supplemental table 3).

10.1136/bmjgh-2024-017484.supp5 Supplementary data

**Table 1:** Percentage distribution of family planning and nutrition-related measures among women of reproductive age (15–49 years) across educational status (prevalence, %, 95% CI), Tanzania Demographic and Health Survey 2022

	Overall	No education	Primary schooling	Middle secondary and higher schooling
Current contraceptive method				
Not using	69.1 (67.9 to 70.3)	76.6 (73.8 to 79.2)	65.9 (64.3 to 67.5)	70.6 (68.8 to 72.3)
Modern methods				
Female sterilisation	2.2 (1.9 to 2.5)	2.5 (1.9 to 3.5)	2.9 (2.5 to 3.4)	0.8 (0.6 to 1.2)
Male sterilisation	0.0 (0.0 to 0.0)	0.0 (0.0 to 0.3)	0.0 (0.0 to 0.1)	0.0 (0.0 to 0.0)
Implants/norplant	11.1 (10.4 to 11.8)	9.4 (7.9 to 11.0)	12.6 (11.6 to 13.7)	9.3 (8.3 to 10.5)
Pill	2.1 (1.8 to 2.4)	1.6 (1.1 to 2.2)	2.5 (2.1 to 3.0)	1.7 (1.3 to 2.2)
Male condom	1.9 (1.6 to 2.2)	0.8 (0.5 to 1.5)	1.6 (1.3 to 2.0)	2.9 (2.4 to 3.5)
Periodic abstinence	3.5 (3.1 to 3.9)	0.7 (0.4 to 1.2)	2.5 (2.1 to 3.0)	6.7 (5.8 to 7.7)
Injections	7.0 (6.4 to 7.6)	6.3 (5.2 to 7.7)	8.6 (7.8 to 9.5)	4.6 (3.8 to 5.4)
IUD	0.6 (0.5 to 0.8)	0.2 (0.0 to 0.4)	0.5 (0.4 to 0.8)	1.0 (0.7 to 1.5)
Emergency contraception	0.0 (0.0 to 0.2)	0.0 (0.0 to 0.0)	0.0 (0.0 to 0.1)	0.2 (0.1 to 0.5)
Female condom	0.0 (0.0 to 0.0)	0.0 (0.0 to 0.0)	0.0 (0.0 to 0.0)	0.0 (0.0 to 0.2)
Traditional methods				
Withdrawal	1.6 (1.4 to 1.9)	1.0 (0.6 to 1.6)	1.8 (1.5 to 2.3)	1.5 (1.2 to 1.9)
Other traditional	0.4 (0.3 to 0.6)	0.4 (0.2 to 0.8)	0.5 (0.3 to 0.7)	0.4 (0.2 to 0.7)
Lactational amenorrhoea	0.4 (0.3 to 0.6)	0.5 (0.3 to 1.1)	0.5 (0.3 to 0.6)	0.3 (0.2 to 0.5)
Standard days method	0.0 (0.0 to 0.0)	0.0 (0.0 to 0.0)	0.0 (0.0 to 0.0)	0.0 (0.0 to 0.0)
Unmet need for family planning*	24.0 (22.8 to 25.2)	29.3†	24.2 (22.7 to 25.8)	19.6 (17.6 to 21.8)
Adolescents 15 to <20 years currently married	11.9 (10.3 to 13.7)	38.6†	17.5†	3.3 (2.4 to 4.7)
Age at first sex				
By age 15 years	13.0 (12.1 to 13.9)	23.0 (21.0 to 25.2)	14.5 (13.4 to 15.7)	5.0 (4.2 to 6.0)
By age 20 years	86.0 (85.0 to 87.0)	95.9 (94.8 to 96.8)	90.4 (89.4 to 91.3)	69.9 (67.8 to 71.9)
Age at first birth				
By age 15 years	2.3 (2.0 to 2.7)	5.6 (4.3 to 7.1)	2.4 (2.0 to 2.8)	0.4 (0.2 to 0.8)
By age 20 years	52.5 (50.9 to 54.2)	67.3 (64.6 to 69.9)	60.2 (58.7 to 61.6)	24.7 (22.8 to 26.7)
Parity				
0	25.4 (24.4 to 26.4)	10.7 (9.3 to 12.2)	17.0 (16.0 to 18.1)	47.6 (45.7 to 49.5)
1	15.0 (14.3 to 15.8)	10.7 (9.2 to 12.4)	13.3 (12.4 to 14.2)	20.3 (19.0 to 21.8)
2–3	28.3 (27.3 to 29.3)	24.6 (22.6 to 26.7)	31.6 (30.3 to 33.0)	24.5 (22.8 to 26.2)
4–5	17.4 (16.5 to 18.2)	23.5 (21.1 to 26.0)	22.0 (20.9 to 23.1)	6.1 (5.2 to 7.2)
6+	13.9 (13.0 to 14.9)	30.6 (28.3 to 33.0)	16.1 (14.9 to 17.4)	1.5 (1.1 to 1.8)
Birth interval				
7–17 months	2.3 (1.9 to 2.7)	2.1†	2.1 (1.6 to 2.6)	3.4 (2.4 to 4.8)
18–23 months	8.5 (7.8 to 9.4)	11.5†	7.7 (6.8 to 8.7)	7.8 (6.3 to 9.6)
24–35 months	38.0 (36.4 to 39.8)	47.5†	37.7 (35.5 to 39.9)	27.2 (24.5 to 30.1)
36–47 months	22.3 (21.2 to 23.4)	21.4†	22.4 (21.1 to 23.8)	22.8 (20.2 to 25.6)
48+ months	28.9 (27.3 to 30.6)	17.5†	30.2 (28.3 to 32.1)	38.8 (35.7 to 42.1)
Anaemia				
Severe	3.1 (2.6 to 3.7)	4.1†	3.2 (2.5 to 4.0)	2.4 (1.8 to 3.2)
Moderate	20.1 (18.9 to 21.4)	22.6†	19.2 (17.7 to 20.8)	20.2 (18.1 to 22.6)
Mild	18.4 (17.2 to 19.6)	17.2†	17.4 (16.1 to 18.9)	20.7 (18.6 to 22.9)
Not anaemic	58.5 (56.7 to 60.2)	56.2†	60.2 (58.1 to 62.2)	56.7 (53.9 to 59.4)
Body mass index				
Underweight	6.0 (5.2 to 6.8)	9.0†	5.3 (4.4 to 6.4)	5.4 (4.4 to 6.7)
Normal	62.8 (61.1 to 64.5)	68.5†	62.9 (60.6 to 65.2)	59.4 (56.9 to 61.9)
Overweight	19.7 (18.3 to 21.0)	16.7†	20.0 (18.4 to 21.7)	20.6 (18.4 to 22.9)
Obese	11.6 (10.6 to 12.8)	5.8†	11.7 (10.3 to 13.2)	14.6 (12.8 to 16.6)

*Unmet need for family planning examined among fecund, sexually active women.

†Estimates of 95% CI not generated as subgroup sample size was insufficient for calculation.

IUD, intrauterine device.

The prevalence of anaemia was about 40% among WRA, with moderate or severe anaemia constituting approximately half of all cases. While the prevalence of underweight was low (6.0%, 95% CI 5.2% to 6.8%), about 30% of WRA were overweight or obese (table 1). Anaemia and underweight prevalence were highest in the lowest education (no education) and wealth (lower quintile) groups, while opposite trends were observed for overweight and obesity (table 1, online supplemental table 2). Anaemia and underweight prevalence were nearly similar in rural and urban areas (online supplemental table 3).

**Figure 1:**
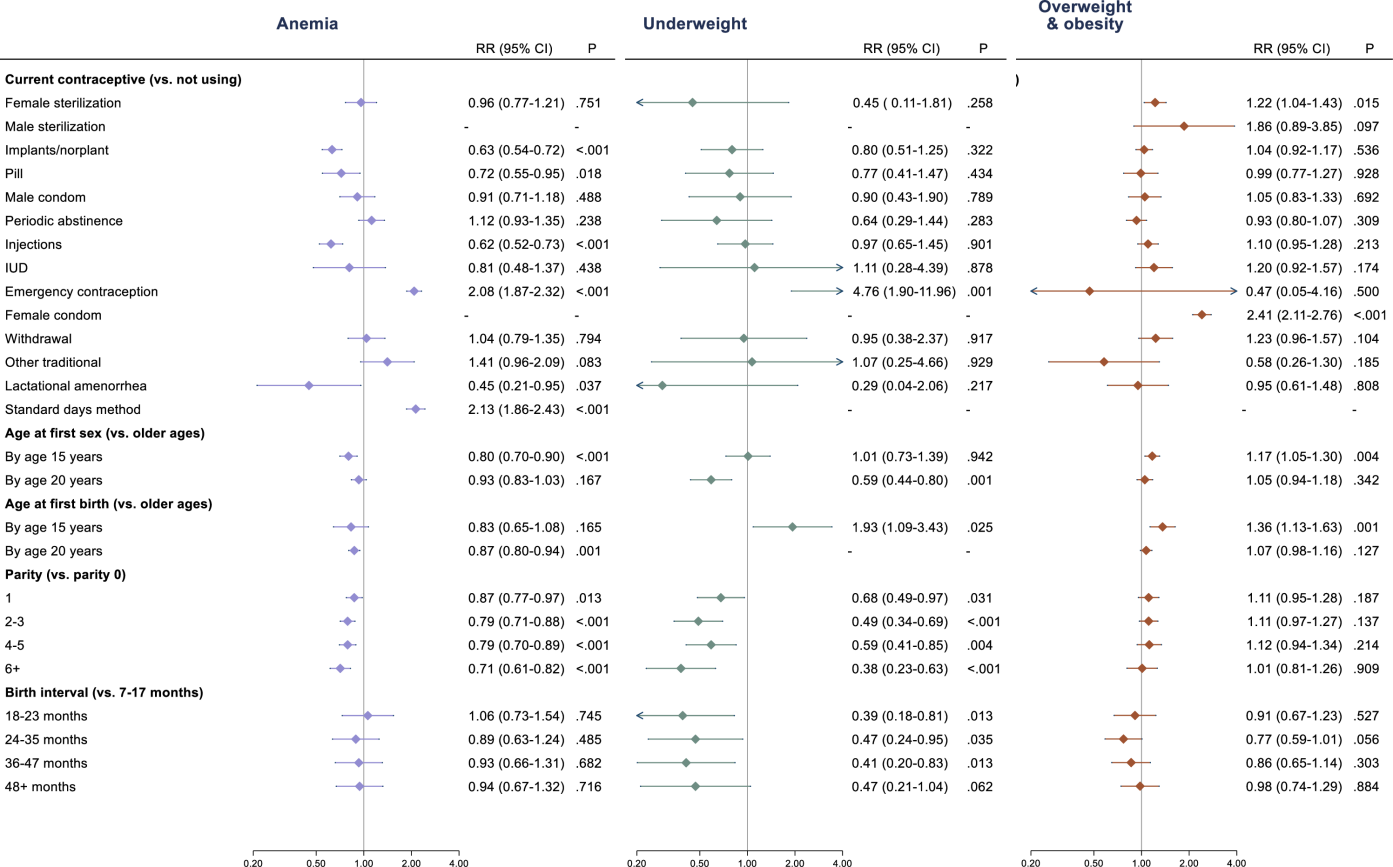
Risk of anemia, underweight and overweight/obesity associated with family planning and related measures among women of reproductive age, Tanzania Demographic and Health Survey 2022. Estimates based on survey-weighted Poisson regression models, adjusted for age category, wealth quintile, education status and rural/urban status. IUD, intrauterine device; RR, risk ratio.

Use of hormonal contraception methods was associated with reduced anaemia risk (risk ratio (RR) for implants: 0.63, 95% CI 0.54 to 0.72, p<0.001; RR for contraceptive pill: 0.72, 95% CI 0.55 to 0.95, p=0.018; RR for injections: 0.62, 95% CI 0.52 to 0.73, p<0.001), while emergency contraception and use of the standard days method were associated with increased risk. Use of female condoms was associated with increased risk of overweight or obesity. A lower risk of being underweight was associated with longer birth spacing intervals (RR for 36–47 vs 1–17 months: 0.41, 95% CI 0.20 to 0.83, p=0.013). Age at first sex by 20 years and parity ≥1 were associated with reduced risk of being underweight, while age at first birth by 15 years was associated with almost twofold increased risk of being underweight (figure 1, online supplemental tables 4 and 5).

### Policy and programme review

We reviewed 25 policy and programme documents^21–45^ using the Walt and Gilson policy triangle to analyse the context, content, processes and actors shaping integrated FP and nutrition in Tanzania, highlighting key implementation challenges and opportunities for strengthening integration. The 25 documents reviewed (2006–2022), most of which were produced between 2010 and 2021, reflect Tanzania’s growing policy attention to FP and nutrition. They include 13 national FP policies and plans, nine national nutrition strategies, four multisectoral documents, two regional and one international partnership report.

While largely health-sector led, several documents demonstrate coordination with agriculture, education and national development sectors through the Prime Minister’s Office. National in scope, they outline goals to increase contraceptive prevalence (27%–28% to 45%–60%), reduce unmet FP need and expand service delivery across facility, community, mobile and digital platforms; nutrition policies target reductions in stunting (42%–24%), undernutrition, anaemia and micronutrient deficiencies while promoting exclusive breastfeeding and addressing rising overweight and obesity. Integrated documents highlight multisectoral and community-engaged approaches to adolescent health, maternal and child nutrition, and reproductive health. Most policies were developed through participatory processes involving government, civil society, development partners, academia and, variably, communities. Online supplemental table 6 provides an overview of these documents, and the following sections present key findings from this review.

#### Context and policy landscape

Tanzania’s FP and nutrition policies are shaped by a dynamic interplay of sociopolitical, economic and international influences, as evidenced in the reviewed documents.^28 35^ These policies have evolved in response to demographic shifts, global health commitments (eg, the 2012 London Summit on Family Planning), and transitions in the country’s political economy,^26 39–41^ factors explicitly cited within the policy texts themselves. This analytical perspective is guided by the Walt and Gilson framework, which emphasises the role of context in policy formulation. For instance, One Plan II (2016), developed as Tanzania moved from low-income to middle-income status, underscores equitable access to quality reproductive, maternal, newborn, child and adolescent health (RMNCAH) services as a foundation for sustainable development. However, Tanzania’s heavy reliance on international donors, driven by commitments such as the 2012 London Summit on Family Planning and FP2020, raises concerns about the long-term sustainability of services.^29^ Short-term funding cycles often hinder continuity, especially in integrated care models. Despite ambitious targets, limited domestic investment and competing priorities challenge the implementation of One Plan II.^18^

Nutrition policy shifts have also responded to rapid population growth, high fertility and the triple burden of malnutrition (ie, under-nutrition, micronutrient deficiencies and overweight/obesity) necessitating integrated health services. Strategic plans like the National Family Planning Costed Implementation Plan (NFPCIP) II and the National Multisectoral Nutrition Action Plan II emphasise integrated service delivery, particularly for women and young children. Yet, existing policies often lack nuanced responses to population diversity across age, geography, ethnicity, education and income as well as guidance on the specificity of integrated services. Cultural and social norms significantly influence policy uptake and hence the National Nutrition Strategy (2012) identified dietary barriers, while NFPCIP II addressed taboos around contraceptive use. These cultural impediments—especially in rural areas—often limit service utilisation and must be explicitly addressed in implementation strategies. Tanzania’s multisectoral policy development process, coordinated by the Prime Minister’s Office, involves various ministries, NGOs and development partners. Policies such as the National Accelerated Action and Investment Agenda for Adolescent Health and Wellbeing illustrate efforts to align local needs with global frameworks. However, our document review found that weak intersectoral collaboration and limited stakeholder engagement are repeatedly cited as hindrances to effective policy implementation within the texts of strategies such as the NFPCIP II and NMNAP II.^28 35^ These documented barriers were corroborated by stakeholders in KIIs, who highlighted fragmented governance and coordination challenges. A persistent shortage of trained healthcare providers, especially in rural areas, also noted in documents like the Human Resource for Health Strategic Plan,^45^ further undermines service integration.

#### Content and programme approaches

The FP policies emphasise postpartum care, access to youth-friendly services and the reduction of contraceptive stockouts. Initiatives include public education campaigns tailored to adolescents and service integration within RMNCAH platforms.^28 39^ However, integration with nutrition remains underdeveloped despite strong conceptual alignment.^22 35 41^

Nutrition strategies, particularly under NMNAP II, focus on human resource development, domestic financing, community engagement and service system strengthening. Proposed integration platforms include health facilities, schools and community outreach, but practical integration with FP services is constrained by separately trained cadres for FP and nutrition. RMNCAH clinics are assumed to offer both services, yet referral systems are unclear, and service guidelines lack specificity.^21 32 35^ Capacity-building efforts are underway, supported by government and international partners.^43^

Some policy documents support integration—for example, the Infant and Young Child Feeding National Guidelines and NMNAP II’s inclusion of FP services as key to nutrition. *“The Lactation Amenorrhea Method (LAM) helps women who wish to use breastfeeding for child spacing…it is advisable for the mother to use another method of family planning to delay pregnancy.”* The Strengthening Postpartum Family Planning and Maternal, Infant, and Young Child Nutrition programme exemplifies full integration of services during pregnancy and the postpartum period, promoting the lactational amenorrhoea method and subsequent transition to modern contraceptives. *“Embedded within Maternal and Child Survival Program’s (MCSP) broader work in Tanzania, this study was designed to assess whether a multi-level facility and community intervention to integrate Maternal, Infant and Young Child Nutrition (MIYCN) and Post Partum Family Planning (PPFP) within existing health contacts in Mara and Kagera contributed to improved service delivery as well as PPFP and MIYCN outcomes.”* However, One Plan II lacks an adaptable framework that reflects Tanzania’s diverse community contexts.

#### Actors and implementation challenges

Multiple actors contribute to policy and programme development. The Ministry of Health and PO-RALG leads planning and implementation, supported by global agencies (WHO, USAID, UNICEF), academic institutions, civil society, the private sector and the media. Despite this broad engagement, fragmentation and role overlap might reduce effectiveness. Local voices are often overshadowed by larger organisations, limiting inclusive, community-responsive policymaking. Implementation frameworks typically span 5–10 years but often lack detailed guidance. For example, the Tanzania Primary Health Services Development Programme uses a hierarchical model, but weak specificity in implementation plans impairs consistency. Interventions are prioritised based on urgency, supported by existing frameworks and monitored using systems like DHIS2 and HMIS. Nonetheless, workforce gaps, fragmented governance and underdeveloped referral pathways impede integration.

In sum, while FP and nutrition services are mutually prioritised in Tanzania’s policies, integration of the two services is constrained by structural, financial and cultural challenges. Discriminatory social norms, inadequate funding, inadequate linkage of FP and nutrition programmes coupled with inadequate coordination and non-communicating monitoring systems are some of the challenges listed in policy and programme documents.^28–30 32–35 41^ Addressing these systemic barriers is essential to realising the potential of integrated service delivery.

### Insights from stakeholder interviews and FGDs

A total of 18 FGDs were conducted between February and May 2024; 9 in each setting, with about 6 participants per group (total=121 participants). In addition, 14 KIIs were conducted with religious leaders, stakeholders from government, NGOs, health sectors and international organisations. FGDs which were done with WRAs, family members and healthcare providers focused on existing practices in healthcare settings around FP and nutrition as well as expected modality and benefits of their integration. KIIs which were conducted with government and non-government actors focused on existing policies and programmes, highlighting gaps and opportunities for integration of the two services. Participants’ characteristics are summarised in table 2.

**Table 2:** Sociodemographic characteristics of KII and FGD Participants

Characteristics	KIIs (n)	FGDs (n)
Total (n)	14	121
Age in years (range)	(27, 69)	(15, 61)
Sex		
Male	6	16
Female	8	105
Education level		
Primary level	–	36
Secondary level	1	67
Tertiary level	13	18
Occupation		
Gynaecologist	1	–
Non-government officials	5	–
Government officials	4	–
Academic Researchers	2	–
Religious leaders	2	–
Nurses	–	6
Community health workers	–	6
Other civil servants	–	8
Entrepreneurs	–	52
Students	–	23
Housewives	–	25

FGD, focus group discussion; KII, key informant interview.

The qualitative findings of group discussion and interviews with stakeholders reveal critical insights into the integration of FP and nutrition services in Tanzania, organised into three overarching themes: contextual needs, service delivery content and platforms, and stakeholder roles and implementation challenges. The themes reflect the challenges and opportunities identified in DHS surveys and national policies, while highlighting critical implementation barriers that hinder progress towards integrated care.

#### Contextual needs: addressing gaps and misconceptions

WRA in Tanzania face a triple burden of malnutrition, including undernutrition, overnutrition and micronutrient deficiencies—alongside persistent unmet FP needs. A representative from the Ministry of Health emphasised, *“When we speak of nutrition* [status of women and children] *in Tanzania, we are talking about a triple burden of undernutrition, micronutrient deficiencies and overweight.”* Low FP uptake remains a concern, with stagnant contraceptive prevalence rates attributed to limited education and cultural barriers. Women in both rural and urban areas face distinct barriers to accessing nutrition and FP services; rural women often contend with long distances and transport costs, while urban women grapple with overcrowded clinics and long wait times, compounding delays in care. There is an urgent need to address these barriers to ensure that women, especially in underserved areas, can access the essential services they need for better health outcomes. Women consistently emphasised that integrating services could reduce these burdens by minimising repeat visits and streamlining care. As women shared, *“When services are offered together, it saves us time and money; especially when we have to walk far or leave our children behind.”*

An NGO official further noted misconceptions such as husbands associating FP with infidelity: *“There are few husbands who believe that once the wife uses family planning, she will be cheating.”* Stigma further complicates access, particularly for unmarried women, as one rural participant shared, *“Being scorned. For example, if she’s unmarried and pregnant, she is treated badly.”* Religious leaders highlighted the potential of faith-based platforms, suggesting, *“Professionals should ask for opportunities in mosques and churches to talk about FP and nutrition.”*

#### Service delivery: fragmentation and emerging integration models

Almost all stakeholders mentioned that FP and nutrition services remain largely siloed, but promising integration points were also identified. WRA in both rural and urban settings expressed that integrated services are vital for addressing their health needs. Women recognise that these services are interconnected, as proper nutrition can improve fertility outcomes, and effective FP enables better spacing of pregnancies, which is crucial for maternal and child health. For instance, a WRA in an urban setting stated, *“It is all about staying healthy because when you combine good nutrition and family planning, you get complete care for your health,”* while an adolescent from a rural setting shared, *“It will help me and other girls like me stay healthier because we will get advice on both nutrition and family planning”*. However, they also described systemic barriers that hinder access. Rural women frequently cited long distances and transport costs, whereas urban women emphasised overcrowded clinics and long wait times. Service delivery challenges such as insufficient trained healthcare workers and supply stockouts were commonly reported across FGDs. On the other hand, healthcare providers mentioned that Reproductive and Child Health Clinics occasionally combine services, as one urban healthcare provider stated, *“When we care for a pregnant mother or a mother seeking birth control, we must touch on nutrition information*.”

Private sector initiatives, such as the *Lishe Endelevu*—Sustainable Nutrition programme, demonstrate successful linkages, with an NGO official stating, *“If a mother breastfeeds exclusively for six months, they automatically have natural birth spacing.”* Participants proposed bundling services; for example, contraceptives with iron supplements and expanding delivery channels to pharmacies, schools and community gatherings. Social media was also highlighted as a key platform, with one NGO official advocating, *“Put it on WhatsApp, Instagram, Twitter, Facebook.”*

#### Stakeholder dynamics and systemic challenges

Successful integration hinges on coordinated stakeholder action and addressing systemic barriers. Women across ages underscored that integration is not just efficient, but it is essential for access and dignity. Rural women emphasised the burden of travelling long distances only to find stockouts or referrals, while urban women described the frustration of navigating overcrowded clinics with fragmented services. As one participant shared, *“Sometimes you wait the whole day, and they say come back tomorrow for family planning—why can’t they do both at once?”* NGOs and donors play a crucial role in filling gaps but expressed concerns about sustainability, noting, *“We need government ownership and buy-in.”* Religious leaders emerged as trusted influencers to combat misconceptions, while academic institutions train providers in disciplinary silos, as one researcher noted, *“We train nutritionists particularly on issues of nutrition and not family planning.”* Fragmented governance, with separate FP and nutrition technical working groups, complicates policy alignment. Resource gaps, including staff shortages and disjointed data systems, further impede progress and reflect the practical aspect of the gaps identified in policies and programme documents. A Ministry of Health representative lamented, *“UCS* [Unified Community System] *data to inform practice gaps is still missing.”* Despite these challenges, opportunities exist through policy alignment and local leadership. An academic researcher emphasised, *“Integration fits existing frameworks,”* while an NGO official stressed the existing alignment at the policy level: *“These services are priority services…they are a priority in the documents. That’s always an opportunity”*

In summary, the qualitative investigation underscored the potential of integrated FP and nutrition services in the context of maternal and child health services to improve health outcomes but emphasises the need for holistic education to counter myths and stigma, structural reforms to unify policies and data systems, and community-centred delivery through faith leaders, schools and digital platforms. Multisectoral collaboration, government commitment and tailored approaches were emphasised to actualise integrated FP and nutrition services for Tanzania’s diverse urban–rural settings. Opportunities for integration include support from local leadership, aligned policies and existence of complementary monitoring and evaluation frameworks.

### Integrated findings: triangulation of quantitative, policy and qualitative evidence

Our mixed-methods design enabled triangulation of findings across data sources, revealing convergence and complementary insights:

1. **Systemic barriers manifest in service delivery:** While policy documents cited ‘fragmented governance’ and ‘siloed programme design’, this was experienced directly by women: *“Sometimes you wait the whole day, and they say come back tomorrow for family planning; why can't they do both at once?”* (Urban WRA, FGD). This explains the quantitative finding that rural women had notably poorer FP indicators, contextualising statistical disparities with lived experience.
2. **Policy-implementation gap:** Despite policy support for integration, stakeholders highlighted implementation failures. A Ministry of Health official noted: *“UCS data to inform practice gaps is still missing”* (KII), while an NGO official observed: *“These services are priority services… they are a priority in the documents. That’s always an opportunity”* (KII), underscoring the chasm between policy intent and operational reality.
3. **FP-nutrition links recognised at multiple levels:** The quantitative association between hormonal contraception and reduced anaemia risk (RR: 0.63 for implants) was echoed in programme logic (eg, *Lishe Endelevu* programme linking breastfeeding with birth spacing) and provider practice: *“When we care for a pregnant mother or a mother seeking birth control, we must touch on nutrition information”* (Healthcare provider, KII).
4. **Context-specific needs require tailored solutions:** Quantitative data showed urban obesity rates were double rural rates (24% vs 12%). Qualitative data explained this through differing barriers: rural women cited ‘long distances and transport costs’, while urban women emphasised ‘overcrowded clinics and long wait times’, demonstrating that integrated models must be contextually adapted.

This integration confirms that statistical trends and policy gaps translate into tangible access barriers, while qualitative data provide the explanatory mechanisms and human dimensions behind the numbers.

## Discussion

This mixed-methods study provides a nuanced understanding of the challenges and opportunities in integrating FP and nutrition services in Tanzania by triangulating quantitative analysis of TDHS 2022 data, desk review of relevant policy and programmes, and qualitative investigation of stakeholder perspective. The findings reveal high unmet needs of FP, high prevalence of anaemia and overweight/obesity among WRA, associations between FP use and nutrition status, critical gaps in integrated service delivery, persistent sociocultural and structural barriers for effective integration, and the potential for integrated approaches to improve health outcomes.

The coexistence of high unmet FP needs and the triple burden of malnutrition, that is, undernutrition, micronutrient deficiencies and overweight/obesity—presents a compelling case for integrated service delivery in Tanzania. Our findings showed that more than two-thirds of Tanzanian WRA were not using contraceptives, with unmet need highest among adolescents and young adults (15–24 years) and women with high parity, mirroring findings from the 1999–2016 TDHS trends^1 50^ and other studies.^51 52^ The concurrent prevalence of anaemia, underweight and overweight and obesity reflects the triple burden of malnutrition reported in the 2018 Tanzania National Nutrition Survey.^2^ This dual burden aligns with regional trends in sub-Saharan Africa, where high fertility and malnutrition coexist due to fragmented health systems and socioeconomic disparities.^14 15^

Notably, hormonal contraceptive use (implants, injections) was associated with reduced anaemia risk, a finding consistent with studies in Burkina Faso and Niger, where integrated FP and iron supplementation improved maternal haemoglobin levels.^53 54^ A study using DHS data from 16 sub-Saharan African countries, conducted between 2015 and 2020, also reported similar findings: women who used hormonal contraceptives were less likely to be anaemic compared with those who did not use them.^55^ This also aligns with physiological evidence: modern contraceptives (eg, injectables, IUDs) reduce menstrual blood loss, a major contributor to iron deficiency in high-fertility settings.^5 55^ Integrated programmes could bundle contraceptives with iron-folic acid supplementation, a strategy being piloted in Ethiopia’s Integrated Family Health Programme.^53 54^

However, the observed association between female condom use and increased overweight/obesity risk, though based on limited data, warrants further investigation, as it contrasts with global evidence suggesting that FP methods generally have neutral or positive effects on nutritional status.^5 56^ Short birth intervals (<24 months) were linked to higher underweight prevalence, reinforcing the need to integrate FP into maternal and child nutrition programmes. Tanzania’s Strengthening Postpartum FP and Nutrition initiative, which promotes the Lactational Amenorrhea Method alongside breastfeeding counselling, demonstrates potential but requires scaling.^17^ Similar integrated postpartum models in Burkina Faso have identified significant gaps, while also identifying strategic opportunities to better align postpartum FP with nutrition and maternal–child health services within routine care systems.^10^

Unmet FP needs were highest among adolescents and rural women, who also face elevated undernutrition. Siloed services exacerbate access barriers; for example, nutrition programmes often target pregnant women, while FP clinics exclude unmarried adolescents due to stigma. Integrated youth-friendly services, like those in Kenya’s Tupange project, could bridge this gap by offering FP and nutrition counselling in schools or community hubs.^14^ While urban women had better FP uptake, obesity prevalence was two times more in rural areas than urban areas. Integrated counselling on healthy spacing and dietary habits, modelled after Bangladesh’s Urban Primary Health Care Project, could address both overweight and obesity and fertility goals.^6 57^

Our policy review revealed that while Tanzania’s One Plan II (2016) and National Nutrition Strategy (2021/2022–2025/2026) advocate for integrated FP and nutrition services, implementation remains limited. Key barriers include siloed programme design, with nutrition and FP operating under separate budgets, training structures and monitoring systems—a challenge similarly noted in Kenya’s RMNCAH programmes.^58^ Donor dependency, driven by short-term funding cycles from initiatives like FP2020, further undermines sustainability, echoing critiques of vertical programming in Uganda and Malawi.^11^ Additionally, while policies such as NMNAP II mention integration, they lack clear operational guidance, including referral mechanisms and joint service delivery protocols, gaps also observed in Ethiopia’s Health Extension Programme.^51^

Stakeholder interviews revealed how policy and system-level gaps manifest in practice. In rural Bagamoyo, stockouts of contraceptives and iron supplements were commonly reported, while urban clinics in Dar es Salaam struggled with overcrowding, often forcing women to choose between FP and nutrition services. These challenges mirror findings from Ghana and Zambia, where fragmented supply chains and workforce shortages hindered integration.^8 9^ Qualitative data also highlighted deeper socio-cultural barriers: rural–urban disparities call for tailored approaches, as seen in Malawi’s Social Cash Transfer Programme^11^; gender norms and misconceptions, such as fears of weight gain or spousal distrust, discouraged FP use^6 15^; and stigma deterred unmarried women from seeking care, echoing adolescent-focused findings from Malawi.^14^

Addressing these barriers will require context-specific strategies, including provider training and community engagement. Stakeholders proposed actionable solutions, including engaging religious leaders to deliver FP-nutrition messages through mosques and churches, an approach shown effective in Senegal’s Muskoka Initiative.^59^ They also recommended integrating FP counselling into community platforms like village meetings, like Ethiopia’s Health Development Army model.^60^ Successful integration requires adapting to Tanzania’s specific context while leveraging global best practices. Key strategies include task-sharing by training community health workers to provide both FP and nutrition services, as seen in Rwanda’s Mutuelle de Santé programme^61^; integrating FP and nutrition indicators within DHIS2 to enable joint outcome tracking, modelled after Malawi’s electronic Health Management Information System (HMIS)^11^; and expanding private sector partnerships through programmes like Lishe Endelevu, which links breastfeeding support with FP, mirroring Bangladesh’s Urban Primary Health Care Project.^5 57^ This finding is also supported by realist evaluations of integrated postpartum projects in Africa, such as the MOMI project, which demonstrated that bundling services and deploying context-specific strategies through community health workers significantly improved care uptake in Burkina Faso, Kenya, Malawi and Mozambique.^62^ Adapting such proven models, particularly those strengthening referral linkages and community-based delivery, could help overcome the systemic fragmentation observed in Tanzania.

This study provides a robust mixed-methods analysis of FP and nutrition integration in Tanzania, drawing on nationally representative 2022 TDHS data, stakeholder interviews from urban and rural settings, and a policy review guided by the Walt and Gilson framework. It offers novel insights into links between contraceptive use and nutritional outcomes and contributes to global evidence on integrated service delivery. Limitations include the cross-sectional design of the quantitative analysis, potential self-reporting bias in the stakeholder interviews, and the limited samples for certain groups such as men, as well as relatively small geographic scope focusing on two sites. Additionally, our quantitative analysis was constrained by the nutrition indicators available in the 2022 TDHS, which focused primarily on anaemia and BMI. The absence of data on specific micronutrient supplementation among all non-pregnant women limited our ability to provide a more comprehensive assessment of women’s nutritional status. We did not examine dietary diversity. Future analyses should consider incorporating broader nutrition metrics to better capture the multifaceted nature of malnutrition and inform integrated programming.

Furthermore, the geographic scope of the qualitative component was limited to two purposively selected wards in Tanzania, ie, one urban and one rural. While this provided valuable contrasting perspectives, the findings may not be fully representative of the diverse contexts across all regions of the country. Also, male participants were underrepresented in the FGDs, which may limit insights into gender dynamics and male perspectives on integrated FP and nutrition services. Still, the study’s methodological depth and triangulation enhance its relevance for informing integration strategies in Tanzania and similar settings.

Tanzania’s intertwined challenges of unmet FP needs and malnutrition demand urgent, systemic action. While this study highlights the promise of integration, from policy alignment to community-driven models, its findings underscore that success hinges on transcending fragmented systems. To realise this potential, Tanzania should prioritise three imperatives: (1) structural reforms to unify FP and nutrition financing, training and monitoring under cohesive governance; (2) context-adaptive delivery that leverages trusted platforms (eg, faith networks, schools and digital tools) to overcome sociocultural barriers and (3) localised evidence generation, including cost-effectiveness analyses and longitudinal studies, to tailor global best practices to Tanzania’s diverse settings. The path forward requires not only government ownership but also bold partnerships across sectors, from frontline health workers to international donors, to transform integration from policy aspiration to lived reality. By addressing these gaps, Tanzania can catalyse a model for integrated care that resonates across sub-Saharan Africa, turning the dual burden of FP and nutrition into an opportunity for holistic health resilience.

## Data Availability

Data are available on reasonable request. Data are available on reasonable request due to privacy/ethical restrictions.
