# Supplementary material for: Opportunities and challenges in integrating family planning and nutrition services in Tanzania: a mixed-methods study
Source: BMJ Glob Health. 2026 Apr 13;10(Suppl 1):e017484. doi: 10.1136/bmjgh-2024-017484 (PMC13158658; doi:10.1136/bmjgh-2024-017484)
Supplement: Supplementary data [file bmjgh-10-Suppl_1-s001.pdf]

Online supplementary File 1. DHS Indicators or Constructed Variables and their Definitions

Variable	Definition or details
Current contraceptive method	Not using, female sterilization, male sterilization, implants/norplant, pill, male condom, periodic abstinence, injections, IUD, emergency contraception, female condom, other modern, withdrawal, other traditional, lactational amenorrhea, standard days method (predefined variable in dataset)
Unmet need for family planning	Among women who are fecund and sexually active (denominator), those who are not using any method of contraception and who report not wanting any more children or wanting to delay the next child (numerator) (1)
Adolescents 15-<20 years currently married	Yes or no (among adolescents only)
Age at first sex by 15 years	Yes or no
Age at first sex by 20 years	Yes or no (among those 20 years and above only)
Age at first birth by 15 years	Yes or no
Age at first birth by 20 years	Yes or no (among those 20 years and above only)
Parity	0, 1, 2-3, 4-5, 6+
Birth interval	Among women with parity of 2 or more, the difference between birth date of two consecutive children, averaged across all births. Categories: 7-17 months, 18-23 months, 24-35 months, 36-47 months, 48+ months
Anemia	No anemia, mild: hemoglobin 11.0-11.9 g/dL, moderate: hemoglobin 8.0-10.9 g/dL, severe: hemoglobin <8.0 g/dL (2) (predefined variable in dataset)
Body mass index (BMI)	Among women aged 20 years and above: underweight: <18.5 kg/m^2, normal: 18.5-24.9 kg/m^2, overweight: 25-29.9 kg/m^2, obese: ≥30 kg/m^2. Among women <20 years of age: underweight: < −2 standard deviations (SD), normal: -2-<1 SD, overweight : 1-<2 SD, obese: ≥2 SD, using the World Health Organization 2007 reference (3,4)
Household has at least one insecticide-treated net	Yes or no
Improved household water source	Improved versus unimproved, as defined by the WHO/UNICEF Joint Monitoring Programme for Water Supply, Sanitation and Hygiene (5)
Improved household toilet facility	Improved versus unimproved, as defined by the WHO/UNICEF Joint Monitoring Programme for Water Supply, Sanitation and Hygiene (6)
Shared household toilet facility	Yes or no
Age category	15-19 years, 20-34 years, 35-49 years
Education	No education, primary, secondary and higher
Wealth quintile	Lowest, lower-middle, middle, upper-middle, highest - taking into account rural/urban status (predefined variable in dataset)
Rural versus urban status	Rural, urban

References for Supplementary File 1

1. Unmet need for family planning (%) [Internet]. [cited 2024 Oct 10]. Available from: https://www.who.int/data/gho/indicator-metadata-registry/imr-details/3414

2. Steele SL, Kroeun H, Karakochuk CD. The Effect of Daily Iron Supplementation with 60 mg Ferrous Sulfate for 12 Weeks on Non-Transferrin Bound Iron Concentrations in Women with a High Prevalence of Hemoglobinopathies. J Clin Med. 2019 Feb 3;8(2):180.

3. de Onis M, Onyango AW, Borghi E, Siyam A, Nishida C, Siekmann J. Development of a WHO growth reference for school-aged children and adolescents. Bull World Health Org. 2007 Sept;85(9):660–7.

4. Obesity and overweight. [Internet]. 2015. Available from: https://www.who.int/en/news-room/fact-sheets/detail/obesity-and-overweight

5. Drinking water | JMP [Internet]. [cited 2024 Oct 10]. Available from: https://washdata.org/monitoring/drinking-water

6. Sanitation | JMP [Internet]. [cited 2024 Oct 10]. Available from: https://washdata.org/monitoring/sanitation
