# Supplementary material for: Opportunities and challenges in integrating family planning and nutrition services in Tanzania: a mixed-methods study
Source: BMJ Glob Health. 2026 Apr 13;10(Suppl 1):e017484. doi: 10.1136/bmjgh-2024-017484 (PMC13158658; doi:10.1136/bmjgh-2024-017484)
Supplement: Supplementary data [file bmjgh-10-Suppl_1-s002.pdf]

Stakeholder perspectives on integrating initiatives in family planning and nutrition
Interview Guide – KIIs (Tanzania and Burkina Faso) Version 1.0 | 05Jul2023

Guide for Key Informant Interviews (KII)

Stakeholder perspectives on integrating initiatives in family planning and nutrition
Interview Guide

Note: Strictly following the guide will not be required, and interviewers may modify the order of overarching themes of questions or adapt/omit/add related questions, based on the participant's background or the information that the participant provides during the interview.

1. **Introduction**

Thank you for agreeing to participate in this interview. As we have mentioned earlier, this study aims to gain perspectives of stakeholders in family planning, nutrition, and related areas to determine whether there are benefits of integrating family planning and nutrition programs. We hope that this work can help to inform appropriate action to improve nutrition and family planning services.

2. **Consent to record the interview**

Before we begin, we would like to ask for your consent to record this interview. Is it okay for us to record this interview?

3. **Participant background details**

We would like to begin by asking you some brief details about yourself. (*The interviewer should ask relevant questions from the form in Appendix III to the participant.)*

4. **Main questions**

Key issues relating to nutrition and family planning

4.1 We would first like to begin with what the key issues related to nutrition are in your country. Could you please outline for us what you think are the main problems or challenges related to nutrition that need to be addressed here?

[*Use probes below if relevant and as appropriate]*

- Could you elaborate further on the scale of these issues?
- How do these issues and challenges have an impact on the population?
- Do you think that these issues affect specific populations in the country more than others?

4.2 We would also like to understand what the key issues related to family planning are in your country. Could you please outline for us what you think are the main problems or challenges related to family planning that need to be addressed here?

[*Use probes below if relevant and as appropriate]*

- Could you elaborate further on the scale of these issues?
- How do these issues and challenges have an impact on the population?
- Do you think that these issues affect specific populations in the country more than others?

Family planning and nutrition programs and policies in the country, including the organization/ institution/ ministry and participant's experience

Laiser Y, *et al. BMJ Glob Health* 2026; 10:e017484. doi: 10.1136/bmjgh-2024-017484

4.3 We would like to know more about your organization/institution/ministry, and the work you do/contribute to in the family planning. What policies and programs does your organization/institution/ministry have in the domain of family planning?

[*Use probes below if relevant and as appropriate]*

- What are the key goals and targets of these policies/programs?
- What is the guiding framework/principles and key activities under these policies/programs?
- How do the existing policies/programs in the country address the needs of vulnerable population, such as adolescents?
- How were these programs/policies formulated?
- Could you please describe how these policies/programs are being implemented?
  - What are the different levels of implementation?
  - Who is involved in the implementation at different levels?
- How are resources allocated for implementation?
- How is the progress of the programs monitored and evaluated?
- We are also interested in understanding the role of stakeholders such as women of reproductive age, religious leaders and other community leaders and members in policies and programs. Can you describe for us how such stakeholders are involved?
  - How are such stakeholders involved during policy/program formulation?
  - How are such stakeholders involved during implementation?
  - How are they involved in tracking progress?
- What are the key successes achieved through these policy and program implementation? Could you share a couple of success stories of implementing these programs and policies?
- What are the key challenges and barriers in implementing the policies and program activities?
  - What steps have been planned or proposed to address these?

4.4 We would like to know more about your organization/institution/ministry, and the work you do/contribute to in nutrition. What policies and programs does your organization/institution/ministry have in the domain of nutrition?

[*Use probes below if relevant and as appropriate]*

- What are the key goals and targets of these policies/programs?
- What is the guiding framework/principles and key activities under these policies/programs?
- How do the existing policies/programs in the country address the needs of vulnerable population, such as adolescents?
- How were these programs/policies formulated?
- Could you please describe how these policies/programs are being implemented?
  - What are the different levels of implementation?
  - Who is involved in the implementation at different levels?
- How are resources allocated for implementation?
- How is the progress of the programs monitored and evaluated?
- We are also interested in understanding the role of stakeholders such as women of reproductive age, religious leaders and other community leaders and members in policies and programs. Can you describe for us how such stakeholders are involved?
  - How are such stakeholders involved during policy/program formulation?
  - How are such stakeholders involved during implementation?
  - How are they involved in tracking progress?

- What are the key successes achieved through these policy and program implementation? Could you share a couple of success stories of implementing these programs and policies?
- What are the key challenges and barriers in implementing the policies and program activities?
  - What steps have been planned or proposed to address these?

<u>Integration of family planning and nutrition policies</u>

4.5 We would like to now discuss with you the current and future opportunities to integrate the family planning and nutrition services in the country. By integration I mean family planning and nutrition services provided at the same healthcare platform or by the same healthcare providers. Sometimes, referral mechanisms might also be established to facilitate the integration of family planning and nutrition services. These efforts might be carried out at the national, regional or pilot-levels.

Would you please tell us about the current integration efforts that are being implemented in the country?
[*Use probes below as appropriate if the participant indicates that such efforts have taken place]*

- What are the motivations for integrating these services?
- What are the expected gains or benefits from integrating these services?
- Who are the key actors involved in planning and implementing this integration?
- How is the integration of family planning and nutrition services taking place?
- What integrated services are provided in your country?
- What is the plan/target for proving these integrated services?
- What delivery platforms are there and who are involved in the delivery of these services?
- What training and capacity building opportunities are needed to ensure that providers have the necessary skills and knowledge to provide integrated family planning and nutrition services?
- How are resources (finance, human, and infrastructure) pulled to facilitate the integration of family planning and nutrition services?
- How are community members and stakeholders engaged in promoting the integration of family planning and nutrition services, and what role do they play in the success of such integration?
- What are the governance mechanisms in place to implement the integrated services (probe on leadership roles and responsibilities, monitoring and evaluation mechanisms) family planning and nutrition services, and how can these be implemented in your setting?
- Can you please share any success stories on the integration of family planning and nutrition services in your country?
  - What do you think may have been the main reasons these were successful?
- Are you aware of any attempts or efforts to integrate family planning and nutrition services that were not successful?
  - What do you think may have been the main reasons these were not successful?
- What are the barriers and challenges to integrating family planning and nutrition services in your area/country?
- How can the integration of family planning and nutrition services be sustained over the long term, and what factors are critical to ensuring its sustainability?

[*Use probes below as appropriate if the participant indicates that such efforts have taken place]*

- Are you aware of any discourse at the national level regarding possible integration of the nutrition and family planning domains?

 - what points have been raised in favour of integrating nutrition and family planning programs?
 - what points have been raised in favour of keeping nutrition and family planning programs separate?
- What do you think are the benefits of integrating family planning and nutrition services?
- What do you think may be some key opportunities in your country to start bringing together services in the domains of family planning and nutrition?
- What integrated services can be provided in your country?
- What delivery platforms can be used and who can be involved in the delivery of these services?
- What training and capacity building opportunities will be needed to ensure that providers have the necessary skills and knowledge to provide integrated family planning and nutrition services?
- How can resources (finance, human, and infrastructure) be pulled to facilitate the integrated services?
- What can be the barriers and challenges to integrating family planning and nutrition services in your area/country?
- Who are the key community members are stakeholders that you think would be important o engaged in this effort to integrate family planning and nutrition services?
  - How can these be engaged effectively?
  - What role can they play in the success of such integration?
- What governance mechanisms can be put in place to implement the integrated services, and how can these be implemented in your setting?
  - What are the key leadership roles that you envision?
  - What would be the other key roles and responsibilities in this governance framework?
  - What sorts of mechanisms for monitoring and evaluation would be important?
- How can the integration of family planning and nutrition services be sustained over the long term, and what factors are critical to ensuring its sustainability?

5. **Additional resources that the participant may know of**

5.1 Are you aware of any documents relevant to family planning, nutrition and integrating family planning and nutrition initiatives, prepared by yourself or others, that you think may be good for us to review?

5.2 Are there any other experts that you recommend we speak with, to gain further perspective in this area?

6. **Closing thoughts and questions**

6.1 Thank you very much for your time speaking with us. It has been very helpful to understand your experiences and perspectives relating to family planning, nutrition and the integration of family planning with nutrition. Are there any additional points that you think would be important for us to consider as we think explore this research question further?

6.2 As we close this interview, do you have any questions for us?

7. **Closing**

Thank you very much once again for your input; it is very much appreciated. Please feel free to get back in touch if you have any further thoughts on this that you would like us to consider. We look forward to remaining in touch.

Stakeholder perspectives on integrating initiatives in family planning and nutrition
Interview Guide – FGDs (Tanzania and Burkina Faso) Version 1.0 | 05Jul2023

Guide for Focus Group Discussion (FGD)

FGD Stakeholder perspectives on integrating initiatives in family planning and nutrition
Interview Guide

NOTE: This guide is worded in a manner targeted for women of reproductive age, and will be adapted as appropriate for other stakeholder groups.

Strictly following the guide will not be required, and interviewers may modify the order of overarching themes of questions or adapt/omit/add related questions, based on the participants' background or the information that the participants provide during the discussion.

1. **Introduction**

Thank you for agreeing to participate in this focus group discussion. As we have mentioned earlier, this study aims to gain perspectives of stakeholders in family planning, nutrition, and related areas to determine whether there are benefits of integrating family planning and nutrition programs. We hope that this work can help to inform appropriate action to improve nutrition and family planning services in this country.

2. **Consent to record the interview**

Before we begin, we would like to ask for your consent to record this interview. Is it okay for us to record this discussion?

3. **Participant background details**

We would like to begin by asking you to introduce yourself. (*The interviewer should then propose on modality that will be used to call on the participants e.g. Using numbers or initials)*

4. **Main questions**

4.1 Family planning and nutrition programs and policies in the country.
Participant's experience in the areas of family planning and nutrition

First, I would like to discuss with you some aspects related to nutrition in your community.

- What are the major health-related concerns among girls/women of your age?
    - Why are these concerns important?
- What are the key nutrition-related problems of women of your age group?
    - Why are these concerns important to address?
- What kind of government-led nutrition services are available for women of your age?
    - Where can women avail these services?
    - Who provides these services?
- What are the challenges in accessing these services?
- How are women involved in providing these services?
- How are women involved in monitoring or evaluating the implementation of services?
- How would you rate the quality of the services?
- What additional services are needed?
    - When, where, and who should provide these additional services?

Laiser Y, *et al. BMJ Glob Health* 2026; 10:e017484. doi: 10.1136/bmjgh-2024-017484

Now I would like to discuss some aspects related to family planning. [*Provide the explanation of family planning that is contextually appropriate*]

- What do you understand by family planning? Why is it important?
- What general practices used to space or limit births or avoid unintended pregnancies by girls/women of your age?
- What kind of government-led healthcare services for family planning are available for women of your age? Where can women avail these services? Who provides these services?
- What are the challenges in accessing these services?
- How are women involved in providing these services?
- How are women involved in monitoring or evaluating the implementation of services?
- How would you rate the quality of the services?
- What additional services are needed?
    - When, where, and who should provide these additional services?

4.2 Integration of family planning and nutrition policies

Now I would like to discuss the current examples where you can access nutrition and family planning services at one place as well as your view on possibly combining these two services.

- What do you think of providing both nutrition-related and family planning-related services together?
- Which services can be provided together? To whom? Why do you think so?
    - [*Note: Probe further for the specific needs of adolescents and young adults, pregnant women attending ANC, Women accessing Postpartum services*]
- What kind of combined services are available for women like you in the health center or in the community? How is this helping you or women like you? Could you elaborate more on it with some examples?
- In the future, if we have to provide nutrition and family planning-related services together, what would be the best ways to do this?
    - Probe: who could provide these services together?
    - Where can be these services be provided?
- What could be the benefits of providing both these services together?
- Who can benefit the most by combining nutrition and family planning related services? How?
- What could be the challenges of providing both these services together?
    - Probe: How can we address these challenges? [*Note: Ask for each challenge*]
- How can we make sure that women of your age are involved in designing the combined services?
- What role you and women like you can play in making sure that these combined services are offered to all women with consistency?
- What role other community members like religious leaders and community leaders can play in making sure that these combined services are offered to all women with consistency?
    - Can you provide some examples?

5 Closing thoughts and questions

5.1 I have these questions for your today. I appreciate all of you sharing your views on these two important topics related to you and women like you. It has been very helpful to understand your experiences and perspectives relating to integration of family planning with nutrition and other

efforts. Are there any additional points that you think would be important for us to consider as we think explore this research question further?

5.2 As we close this discussion, do you have any questions for us?

6 Closing

Thank you very much once again for your input; it is very much appreciated. Please feel free to get back in touch if you have any further thoughts on this that you would like us to consider. We look forward to remaining in touch.
