# Supplementary material for: Opportunities and challenges in integrating family planning and nutrition services in Tanzania: a mixed-methods study
Source: BMJ Glob Health. 2026 Apr 13;10(Suppl 1):e017484. doi: 10.1136/bmjgh-2024-017484 (PMC13158658; doi:10.1136/bmjgh-2024-017484)
Supplement: Supplementary data [file bmjgh-10-Suppl_1-s003.pdf]

Online Supplemental File 1: Reflexivity Statement

1. How does this study address local research and policy priorities?

This study responds to Tanzania's pressing research and policy needs by focusing on the integration of family planning, nutrition, and broader sexual and reproductive health services as areas that are increasingly recognized for their essential role in improving health outcomes among women of reproductive age. By identifying existing gaps and uncovering opportunities within current service delivery models, the study contributes to the national dialogue on how best to enhance health system efficiency and effectiveness. Building on insights from multiple stakeholders and evidence from existing family planning and nutrition documents, the research aims to generate context-specific recommendations that can inform future programming and guide policy decisions to better serve communities across Tanzania and other Low- and Middle-Income Countries (LMIC).

2. How were local researchers involved in study design?

The research team brings together a diverse group of experienced researchers with deep roots in public health and qualitative research in Tanzania and across LMICs. MS, IY, YL, IK and JH have extensive experience conducting public health research in Tanzania, while SS and UP are originally from LMICs and currently based in high-income countries. WF and IS have led or supported numerous global research initiatives involving LMICs, further enriching the team's collective expertise. All authors were actively involved in shaping and refining the study design. Our team represents a rich blend of cultural backgrounds and lived experiences, which strengthens the relevance and sensitivity of our work. We are firmly committed to advancing equity, promoting inclusive engagement, and ensuring genuine collaboration at every stage of the research process.

3. How has funding been used to support the local research team?

Funding has been used to support the local research team by covering costs related to participant recruitment, data collection, and analysis, thereby enhancing their capacity to conduct high-quality research. Funding has also enabled the local research team to attend conferences and workshops and meet with other local researchers, policymakers and program leaders (including those from international organizations in Tanzania) to present preliminary findings from this study and other studies within the broader project – aiding to broaden scientific and programmatic networks and strengthen partnerships between the local research teams and other relevant local stakeholders.

4. How are research staff who conducted data collection acknowledged?

YL, IK, JH and IY conducted data collection, and are included as authors. SS, UP and IS contributed in analysis and every team member was designated and delivered specific role(s) during the writing process.

5. Do all members of the research partnership have access to study data?

In line with participant consent, only the PIs (WF&MS) and a restricted set of individuals in the study team who required access to the data for analytical purposes (including co-authors of this manuscript) have access to the data.

6. How was data used to develop analytical skills within the partnership?

All the authors listed on this manuscript were involved in all the stages of research, with intentions to utilize multidisciplinary skills and develop accessible language for a broad research audience.

7. How have research partners collaborated in interpreting study data?

Three workshops were held during the analysis stage. The first two days defined research, getting familiar with the data and data extraction sheets. Researchers then formed working groups to conduct analysis and interpretation and further discussed the findings collectively, which were reported at a subsequent workshop where recommendations and reflexivity statements were finalized collaboratively.

8. How were research partners supported to develop writing skills?

The research team drafting this statement is primarily made up of senior academics. Early career researchers (SS, UP, YL, IY, YZ, IK and JH) received support from senior academics (IS, MS and WF) to develop and refine their writing skills.

9. How will research products be shared to address local needs?

This manuscript will be published as a part of a Special Issue on integration of family planning and nutrition services and as an open access. We plan to disseminate this issue widely, including outreach to global health leaders, international collaboration experts, and other stakeholders in both high- and low- and middle-income countries.

10. How is the leadership, contribution and ownership of this work by LMIC researchers recognized within the authorship?

Authors YL and IY from Tanzania, contributed as joint first authors in developing this manuscript, working alongside the senior authorship team. UP and SS, also originally from LMICs, contributed significantly and are co-authors. The team comprises a well-balanced mix of researchers based in both high-income and low- and middle-income countries, fostering a collaborative approach that integrates diverse perspectives and experiences.

11. How have early career researchers across the partnership been included within the authorship team?

We have included early career researchers (SS, UP, YL, IY, YZ, IK and JH) within the authorship team. They contributed to multiple stages of this research.

12. How has gender balance been addressed within the authorship?

Five authors are male (SS, YL, IY, IS and WF) and five authors are female (UP, IK, JI, YZ and MS); all the female authors were actively engaged in conceiving the study, data collection, data analysis and interpretation.

13. How has the project contributed to training of LMIC researchers?

The authorship team includes both early-career and senior researchers. Research funding supported the employment of a junior researcher in Tanzania.

14. How has the project contributed to improvements in local infrastructure?

This project has not directly contributed to improvements in local infrastructure.

15. What safeguarding procedures were used to protect local study participants and researchers?

We implemented stringent safeguarding procedures, including ethical review and informed consent processes, to protect both local study participants and researchers. Regular monitoring and support procedures were implemented to ensure adherence to these protocols.

16. How did the research team's positionalities, values, and assumptions shape the study, and how were these influences managed?

Our shared commitment to equity, interdisciplinary integration, and evidence informed policy likely shaped the research by foregrounding systemic gaps and favoring integrated, community centered solutions in our analysis. This lens may have inclined us toward interpreting data through a framework of fragmentation and missed opportunities for synergy. To mitigate this potential bias and ensure our conclusions remained grounded in the data, we employed several strategies. We maintained a diverse team encompassing Tanzanian and international perspectives, researchers and practitioners. We used structured analytical frameworks, such as the Walt and Gilson policy triangle, to guide objective data extraction and analysis for the desk review while systematic thematic analysis for the qualitative data generated through interviews and focus group discussions. Finally, we held regular consensus discussions where interpretations were explicitly challenged, and findings were rigorously cross checked against the raw data, i.e., quantitative statistics, direct policy text, and verbatim participant quotes, to separate our advocacy-oriented values from the evidence generated by the study.
