# Supplementary material for: Opportunities and challenges in integrating family planning and nutrition services in Tanzania: a mixed-methods study
Source: BMJ Glob Health. 2026 Apr 13;10(Suppl 1):e017484. doi: 10.1136/bmjgh-2024-017484 (PMC13158658; doi:10.1136/bmjgh-2024-017484)
Supplement: Supplementary data [file bmjgh-10-Suppl_1-s004.pdf]

Supplementary File 4

GRAMMS - O'Cathain A, Murphy E, Nicholl J. The quality of mixed methods studies in health services research. J Health Serv Res Policy. 2008;13(2):92-98

Reporting Item	Where in Manuscript
(1) Describe the justification for using a mixed methods approach to the research question	**Methods pages 8-9 and 13-14**: This mixed-methods study was conducted with concurrent quantitative and qualitative approaches. A mixed-methods approach was essential to fully investigate the integration of FP and nutrition services, enabling us to statistically quantify population-level links, critically analyze supportive yet inadequately implemented policies, and understand the real-world barriers and opportunities for integrated service delivery from the perspective of stakeholders and communities.
(2) Describe the design in terms of the purpose, priority and sequence of methods	**Methods page 13-14**: This mixed-methods study was conducted with concurrent quantitative and qualitative approaches.
(3) Describe each method in terms of sampling, data collection and analysis	**Methods**:<br><br>**Quantitative (page 9)**<br>Sample: TDHS 2022 Survey Analysis:<br>descriptive statistics and regression analysis<br><br>**Qualitative (page 10-13)**<br>Purposive sampling<br>Data collection: one-on-one semi- structured interviews and focus group discussions<br>Thematic analysis<br><br>**Desk Review (page 10)**<br>Systematic search of documents<br>Thematic analysis
**(4)** Describe where integration has occurred, how **it** has occurred and who has participated in it	**Methods page 13-14**: We have demonstrated triangulation through a dedicated results subsection that explicitly compares and synthesizes quantitative trends, policy gaps, and qualitative narratives to reveal convergence and complementary insights across all three data sources.
(5) Describe any limitation of one method associated with the present of the other method	Interpretation in **Discussion, page 33**: The cross-sectional nature of the quantitative survey limits causal inference, while the qualitative findings provide contextual depth but cannot statistically generalize the observed associations.

(6) Describe any insights gained from mixing or integrating methods	Conclusion in **Results, page 27-28 and Discussion, page 28-29 and 33**: Based on our mixed methods findings, we conclude that while a strong policy foundation and clear biological links exist for integrating family planning and nutrition services in Tanzania, this potential is unrealized due to persistent systemic fragmentation. Successful integration requires moving beyond conceptual alignment to actionable implementation. This entails unified governance to synchronize planning and budgets, context adaptive delivery models that bundle services and leverage trusted community platforms, and strengthened health systems through integrated training, supply chains, and data monitoring. Prioritizing underserved groups such as rural women and adolescents and securing sustainable domestic financing are critical. By addressing these gaps, Tanzania can transform its dual burden of unmet FP needs and malnutrition into an opportunity for holistic, resilient maternal and child health, offering a replicable model for similar settings across sub-Saharan Africa.

Tong A, Sainsbury P, Craig J. Consolidated criteria for reporting qualitative research {COREQ): a 32-item checklist for interviews and focus groups. Int J Qual Health Care. 2007;19(6) :349-357.

No	Personal Characteristics	Guide questions/description	Where in Manuscript
I Domain 1: Research team and reflexivity			
I Personal Characteristics			
1	Interview er/facilitator	Which author/s conducted the interview or focus group?	The FGDs were facilitated by two trained female research assistants with bachelor’s degrees in social sciences or public health, supervised by senior researchers (YL, IY). The KIIs were conducted by two senior researchers (YL, IY) with advanced degrees and extensive expertise in public health and qualitative methods. The study team comprised multidisciplinary researchers with expertise in public health, nutrition, family planning, qualitative and quantitative methods, and implementation research, with members based in both Tanzania and international institutions. **(page 10-13)**
2	Credentials	What were the researcher's credentials? E.g. PhD, MD	
3	Occupation	What was their occupation at the time of the study?	
4	Gender	Was the researcher male or female?	
5	Experience and training	What experience or training did the researcher have?	
Relationship with participants			
6	Relationship established	Was a relationship established prior to study commencement?	No. Methods: There were no pre- existing relationships with study participants and non-participation was not tracked. **(page 10-13)**
7	Participant knowledge of the interviewer	What did the participants know about the researcher? e.g. personal goals, reasons for doing the research	Yes - informed consent<br>See appended interview and focus group discussion guides **(page 10-13, Online supplementary file 2**)
8	Interviewer characteristics	What characteristics were reported about the interviewer/facilitator? e.g. Bias, assumptions, reasons and interests in the research topic	Methods: Our multidisciplinary team, with expertise in global health, public health, nutrition, and sexual and reproductive health, conducted the research; we acknowledged that our shared commitment to equity and integrated solutions may have shaped the study's focus, and we managed potential bias through diverse team composition, structured frameworks, and consensus-based analysis. **(page 14)**

9	Methodological orientation and Theory	What methodological orientation was stated to underpin the study? e.g. grounded theory, discourse analysis, ethnography, phenomenology, content analysis	Methods: A hybrid inductive-deductive thematic analysis **(page 10-13)**
Participant Selection			
10	Sampling	How were participants selected? e.g. purposive, convenience, consecutive, snowball	Methods: Purposive **(page 10-13)**
11	Method of approach	How were participants approached? e.g. face-to-face, telephone, mail, email	Methods: Face-to-face **(page 10-13)**
12	Sample size	How many participants were in the study?	Results: A total of 121 participants took part in 18 Focus Group Discussions, and an additional 14 participants were engaged through Key Informant Interviews. Further details on participant characteristics, including age, sex, education, and occupation, are provided in **Table 2 (page 23).**
13	Non-participation	How many people ref used to participate or dropped out? Reasons?	Methods: There were no pre-existing relationships with study participants and non-participation was not tracked. **(page 10-13)**
Setting			
14	Setting of data collection	Where was the data collected? e.g. home, clinic, workplace	Methods: In-person **(page 10-13)**
15	Presence of non-participants	Was anyone else present besides the participants and researchers?	No. Methods: one-on-one semi- structured interviews or focus group discissions **(page 10-13)**
16	Description of sample	What are the important characteristics of the sample? e.g. demographic data, date Data collection	Table 2, **Page 23**

I Data Collection

17	Interview guide	Were questions, prompts, guides provided by the authors? Was it pilot tested?	See appended interview and focus group discussion guides (**Online supplementary File 2)**<br><br>Methods: authors reviewed the developed interview guides **(page 10-13)**
18	Repeat interviews	Were repeat interviews carried out? If yes, how many?	Not applicable
19	Audio/visual recording	Did the research use audio or visual recording to collect the data?	Methods: We audio-recorded interviews. **(page 10-13)**
20	Field notes	Were field notes made during and/or after the interview or focus group?	Yes. Methods: Throughout we engaged in verification strategies including field notes and frequent team debriefings before, during and after analysis. **(page 10-13)**
21	Duration	What was the duration of the interviews or focus group?	Methods: Interviewers and group discussion facilitators transcribed and translated interviews and discussions; ranging between 45-75 minutes. **(page 10-13)**
22	Data saturation	Was data saturation discussed?	Methods: Recruitment stopped once purposeful sampling was completed. **(page 10-13)**
23	Transcripts returned	Were transcripts returned to participants for comment and/or correction?	Methods: No. Transcripts were cleaned to ensure de-identification and checked for quality/accuracy by the study team. **(page 10-13)**

I Data Analysis

24	Number of data coders	How many data coders coded the data?	Methods: Three authors coded all transcripts; the research team check the coded data to test the fit and relevance and reviewed the coding. Minor revisions were made during pilot coding to the codebook for conceptual fit. **(page 10-13)**
25	Description of the coding tree	Did authors provide a description of the coding tree?	Methods: Coding tree is not described in its entirety; description of emerging themes relevant to this manuscript is provided **(page 10-13)**
26	Derivation of themes	Were themes identified in advance or derived from the data?	Methods: A combination of inductive and deductive approach to thematic analysis was used. **(page 10-13)**

27	Software	What software, if applicable, was used to manage the data?	Methods: Microsoft Excel was used to code data **(page 10-13)**
28	Participant checking	Did participants provide feedback on the findings?	No. There were no pre-existing partnerships with study participants, and we did not approach participants for feedback on the findings. **(page 10-13)**
Reporting			
29	Quotations presented	Were participant quotations presented to illustrate the themes/ findings? Was each quotation identified? e.g. participant number	Yes, participant quotes are used throughout the qualitative study findings. No participant number is assigned. (**page 23-27**)
30	Data and findings consistent	Was there consistency between the data presented and the findings?	Yes, there was consistency between the data presented and the findings; all conclusions are directly supported by the reported quantitative results and qualitative evidence, with no discrepancies between the data shown and the interpretations made. (**page 23-27**)
31	Clarity of major themes	Were major themes clearly presented in the findings?	Major themes emerging from the desk review and qualitative investigation are presented under sub-headings. (**pages 18-22 for desk review, and 23-27 for qualitative findings**)
32	Clarity of minor themes	Is there a description of diverse cases or discussion of minor themes?	Yes, where appropriate minor themes are described and discussed. (**page 23-27**)
