# Supplementary material for: Opportunities and challenges in integrating family planning and nutrition services in Tanzania: a mixed-methods study
Source: BMJ Glob Health. 2026 Apr 13;10(Suppl 1):e017484. doi: 10.1136/bmjgh-2024-017484 (PMC13158658; doi:10.1136/bmjgh-2024-017484)
Supplement: Supplementary data [file bmjgh-10-Suppl_1-s005.pdf]

Supplementary Table 1. Distribution of family planning and nutrition related measures among women of reproductive age overall and across age groups (prevalence, %, 95% confidence interval), Tanzania Demographic and Health Survey 2022.

	Overall	15-19 years	20-34 years	35-49 years
**Current contraceptive method**				
**Not using**	69.1 (67.9-70.3)	91.8 (90.4-93.0)	63.0 (61.2-64.6)	63.9 (62.0-65.8)
**Modern methods**				
**Female sterilization**	2.2 (1.9-2.5)	0.0 (0.0-0.0)	0.3 (0.2-0.6)	6.5 (5.7-4.4)
**Male sterilization**	0.0 (0.0-0.0)	0.0 (0.0-0.0)	0.0 (0.0-0.0)	0.1 (0.0-0.3)
**Implants/norplant**	11.1 (10.4-11.8)	3.3 (2.5-4.2)	15.1 (14.0-16.3)	10.0 (9.0-11.1)
**Pill**	2.1 (1.8-2.4)	0.2 (0.0-0.4)	2.6 (2.1-3.1)	2.6 (2.1-3.2)
**Male condom**	1.9 (1.6-2.2)	1.6 (1.1-2.3)	2.2 (1.8-2.7)	1.6 (1.2-2.1)
**Periodic abstinence**	3.5 (3.1-3.9)	1.1 (0.6-1.8)	4.1 (3.5-4.7)	4.2 (3.5-5.0)
**Injections**	7.0 (6.4-7.6)	1.4 (1.0-2.0)	9.0 (8.1-10.0)	7.5 (6.6-8.5)
**IUD**	0.6 (0.5-0.8)	0.0 (0.0-0.2)	0.7 (0.5-1.1)	0.9 (0.6-1.2)
**Emergency contraception**	0.0 (0.0-0.2)	0.0 (0.0-0.0)	0.2 (0.0-0.4)	0.0 (0.0-0.0)
**Female condom**	0.0 (0.0-0.0)	0.0 (0.0-0.0)	0.0 (0.0-0.0)	0.0 (0.0-0.0)
**Traditional methods**				
**Withdrawal**	1.6 (1.4-1.9)	0.5 (0.3-0.9)	1.8 (1.5-2.3)	1.9 (1.5-2.4)
**Other traditional**	0.4 (0.3-0.6)	0.0 (0.0-0.3)	0.4 (0.3-0.7)	0.7 (0.4-1.0)
**Lactational amenorrhea**	0.4 (0.3-0.6)	3.3 (2.5-4.2)	0.6 (0.4-0.8)	0.3 (0.2-0.5)
**Standard days method**	0.0 (0.0-0.0)	0.0 (0.0-0.0)	0.0 (0.0-0.0)	0.0 (0.0-0.0)
**Unmet need for family planning[1]**	24.0 (22.8-25.2)	31.5*	22.3 (21.0-23.7)	25.0 (23.0-27.0)
**Adolescents 15-<20 years currently married**	11.9 (10.3-13.7)	11.9 (10.3-13.7)	-	-
**Age at first sex**				
**By age 15 years**	13.0 (12.1-13.9)	8.7 (7.5-10.2)	13.8 (12.7-14.9)	14.5 (13.1-16.0)
**By age 20 years**	86.0 (85.0-87.0)	-	83.4 (82.1-84.7)	86.4 (85.0-87.7)
**Age at first birth**				
**By age 15 years**	2.3 (2.0-2.7)	0.9 (0.6-1.4)	2.3 (1.9-2.7)	3.2 (2.5-4.0)
**By age 20 years**	52.5 (50.9-54.2)	-	49.6 (47.7-51.6)	52.1 (50.0-54.2)
**Parity**				
**0**	25.4 (24.4-26.4)	84.0 (82.1-85.7)	15.5 (14.2-16.8)	3.1 (2.5-3.8)
**1**	15.0 (14.3-15.8)	13.6 (12.1-15.4)	22.4 (21.2-23.7)	4.7 (4.0-5.5)
**2-3**	28.3 (27.3-29.3)	2.4 (1.8-3.1)	42.0 (40.6-43.5)	24.0 (22.1-26.0)
**4-5**	17.4 (16.5-18.2)	0.0 (0.0-0.0)	15.8 (14.7-17.0)	30.7 (28.8-32.6)
**6+**	13.9 (13.0-14.9)	0.0 (0.0-0.0)	4.2 (3.6-5.0)	37.5 (35.2-40.0)
**Birth interval**				
**7-17 months**	2.3 (1.9-2.7)	14.8*	3.4 (2.8-4.3)	0.9 (0.6-1.3)
**18-23 months**	8.5 (7.8-9.4)	20.6*	10.9 (9.8-12.0)	5.9 (5.1-7.0)
**24-35 months**	38.0 (36.4-39.8)	53.6*	37.9 (35.9-39.9)	38.0 (35.8-40.2)
**36-47 months**	22.3 (21.2-23.4)	8.4*	21.9 (20.5-23.5)	22.8 (21.2-24.5)
**48+ months**	28.9 (27.3-30.6)	2.6*	25.9 (24.0-27.9)	32.4 (30.4-34.4)
**Anemia**				
**Severe**	3.1 (2.6-3.7)	1.9 (1.3-2.7)	3.1 (2.4-4.1)	3.8 (2.9-5.0)
**Moderate**	20.1 (18.9-21.4)	21.1 (18.4-24.2)	19.9 (18.2-21.7)	20.0 (17.8-21.7)

Laiser Y, *et al. BMJ Glob Health* 2026; 10:e017484. doi: 10.1136/bmjgh-2024-017484

Supplementary Table 1. Distribution of family planning and nutrition related measures among women of reproductive age overall and across age groups (prevalence, %, 95% confidence interval), Tanzania Demographic and Health Survey 2022.

	Overall	15-19 years	20-34 years	35-49 years
**Mild**	18.4 (17.2-19.6)	22.0 (19.6-24.7)	18.0 (16.6-19.6)	16.4 (14.6-18.4)
**Not anemic**	58.5 (56.7-60.2)	55.0 (51.6-58.4)	59.0 (56.5-61.3)	60.1 (57.5-62.6)
**BMI**				
**Underweight**	6.0 (5.2-6.8)	3.2 (2.3-4.6)	7.8 (6.7-9.1)	4.9 (3.9-6.1)
**Normal**	62.8 (61.1-64.5)	84.0 (81.7-86.0)	61.6 (59.2-63.9)	50.5 (47.8-53.3)
**Overweight**	19.7 (18.3-21.0)	10.3 (8.6-12.3)	20.1 (18.2-22.1)	25.2 (22.8-27.7)
**Obese**	11.6 (10.6-12.8)	2.5 (1.6-3.8)	10.6 (9.3-12.0)	19.4 (17.2-21.9)

[1]Unmet need for family planning examined among fecund, sexually active women.

*Estimates of 95% confidence interval not generated as subgroup sample size was insufficient for calculation.

Supplementary Table 2. Distribution of family planning and nutrition related measures among women of reproductive age across wealth quintile (prevalence, 95% confidence interval), Tanzania Demographic and Health Survey 2022.

	Lowest	Lower-middle	Middle	Upper-middle	Highest
**Current contraceptive method**					
**Not using**	75.1*	70.7 (68.1-73.2)	67.7 (65.5-69.9)	66.2 (64.2-68.2)	67.1*
**Modern methods**					
**Female sterilization**	1.6*	2.2 (1.6-3.0)	1.6 (1.2-2.2)	2.5 (2.0-3.2)	2.8*
**Male sterilization**	0.0*	0.0 (0.0-0.3)	0.0 (0.0-0.0)	0.0 (0.0-0.3)	0.0*
**Implants/norplant**	10.9*	11.5 (10.2-13.0)	11.8 (10.4-13.4)	11.9 (10.5-13.5)	9.6*
**Pill**	1.5*	2.1 (1.5-2.9)	2.8 (2.1-3.6)	2.4 (1.8-3.1)	1.7*
**Male condom**	1.2*	1.6 (1.1-2.3)	1.5 (1.1-2.2)	2.3 (1.7-3.1)	2.6*
**Periodic abstinence**	1.2*	1.9 (1.4-2.6)	2.6 (2.0-3.4)	4.2 (3.3-5.3)	6.5*
**Injections**	5.7*	7.7 (6.5-9.0)	8.8 (7.4-10.5)	7.0 (6.0-8.1)	5.9*
**IUD**	0.2*	0.3 (0.1-0.6)	0.5 (0.3-0.8)	0.7 (0.4-1.2)	1.4*
**Emergency contraception**	0.0*	0.0 (0.0-0.0)	0.0 (0.0-0.0)	0.0 (0.0-0.3)	0.2*
**Female condom**	0.0*	0.0 (0.0-0.0)	0.0 (0.0-0.0)	0.0 (0.0-0.1)	0.0*
**Traditional methods**					
**Withdrawal**	1.5*	1.2 (0.7-1.8)	1.8 (1.3-2.6)	1.9 (1.4-2.6)	1.6*
**Other traditional**	0.4*	0.4 (0.2-0.8)	0.4 (0.2-0.8)	0.6 (0.3-1.2)	0.3*
**Lactational amenorrhea**	0.7*	0.4 (0.2-0.9)	0.4 (0.2-0.8)	0.3 (0.1-0.5)	0.3*
**Standard days method**	0.0*	0.0 (0.0-0.0)	0.0 (0.0-0.0)	0.0 (0.0-0.0)	0.0*
**Unmet need for family planning[1]**	27.8*	27.1 (24.7-29.7)	23.5 (21.3-25.9)	22.4 (20.4-24.4)	19.8*
**Adolescents 15-<20 years currently married**	19.7*	15.6*	13.6*	8.7*	5.0*
**Age at first sex**					
**By age 15 years**	17.9*	17.1 (15.3-19.2)	12.7 (11.2-14.4)	10.8 (9.5-12.1)	8.2*
**By age 20 years**	94.0*	91.8 (90.5-93.0)	87.6 (85.9-89.2)	85.3 (83.6-86.9)	74.4*
**Age at first birth**					
**By age 15 years**	3.1*	3.0 (2.4-3.9)	1.9 (1.4-2.6)	1.9 (1.3-2.6)	1.8*
**By age 20 years**	63.1*	58.3 (55.7-60.9)	54.9 (52.3-57.4)	51.4 (48.5-54.3)	38.7*
**Parity**					
**0**	19.4*	21.7 (19.8-23.7)	24.6 (22.7-26.6)	25.5 (23.7-27.3)	33.3*
**1**	12.4*	14.8 (13.3-16.6)	15.8 (14.1-17.5)	15.2 (13.9-16.6)	16.3*
**2-3**	26.6*	28.8 (26.6-31.2)	27.8 (25.8-29.9)	29.2 (27.1-31.4)	28.8*
**4-5**	20.5*	18.0 (16.5-19.7)	17.5 (16.0-19.1)	17.1 (15.7-18.6)	14.6*
**6+**	21.1*	16.6 (15.0-18.3)	14.4 (12.8-16.0)	13.1 (11.6-14.8)	7.1*
**Birth interval**					
**7-17 months**	1.6*	2.5*	2.7 (1.9-3.8)	2.0 (1.3-2.9)	2.7*
**18-23 months**	11.0*	9.6*	8.0 (6.6-9.7)	7.1 (5.3-9.5)	7.0*
**24-35 months**	48.1*	41.6*	38.3 (34.9-41.8)	34.2 (31.2-37.2)	28.3*
**36-47 months**	20.8*	22.4*	22.3 (20.1-24.6)	21.8 (19.5-24.3)	24.0*
**48+ months**	18.5*	24.0*	28.7 (25.9-31.7)	35.0 (32.3-37.8)	38.0*
**Anemia**					

Supplementary Table 2. Distribution of family planning and nutrition related measures among women of reproductive age across wealth quintile (prevalence, 95% confidence interval), Tanzania Demographic and Health Survey 2022.

	Lowest	Lower-middle	Middle	Upper-middle	Highest
**Severe**	3.6*	3.3*	2.1*	3.3*	3.2*
**Moderate**	22.3*	21.5*	18.7*	18.6*	19.9*
**Mild**	18.9*	17.8*	16.7*	18.2*	20.0*
**Not anemic**	55.3*	57.3*	62.4*	60.0*	57.0*
**BMI**					
**Underweight**	8.5*	7.7*	5.1*	4.7*	4.6*
**Normal**	72.4*	66.2*	64.6*	61.9*	52.1*
**Overweight**	15.1*	17.9*	20.1*	20.4*	23.3*
**Obese**	4.0*	8.3*	10.2*	13.1*	20.0*

[1]Unmet need for family planning examined among fecund, sexually active women.

*Estimates of 95% confidence interval not generated as subgroup sample size was insufficient for calculation.

Laiser Y, *et al. BMJ Glob Health* 2026; 10:e017484. doi: 10.1136/bmjgh-2024-017484

Supplementary Table 3. Distribution of family planning and nutrition related measures among women of reproductive age across urban/rural status (prevalence, %, 95% confidence interval), Tanzania Demographic and Health Survey 2022.

	Urban	Rural
**Current contraceptive method**		
**Not using**	64.8*	71.5 (70.0-72.9)
**Modern methods**		
**Female sterilization**	2.1*	2.3 (1.9-2.6)
**Male sterilization**	0.0*	0.0 (0.0-0.1)
**Implants/norplant**	11.2*	11.0 (10.1-12.0)
**Pill**	2.4*	1.9 (1.6-2.3)
**Male condom**	2.8*	1.4 (1.1-1.7)
**Periodic abstinence**	6.3*	2.0 (1.6-2.3)
**Injections**	6.6*	7.2 (6.5-8.0)
**IUD**	0.8*	0.5 (0.4-0.8)
**Emergency contraception**	0.2*	0.0 (0.0-0.1)
**Female condom**	0.0*	0.0 (0.0-0.0)
**Traditional methods**		
**Withdrawal**	2.1*	1.3 (1.1-1.7)
**Other traditional**	0.4*	0.4 (0.3-0.6)
**Lactational amenorrhea**	0.4*	0.4 (0.3-0.6)
**Standard days method**	0.0*	0.0 (0.0-0.0)
**Unmet need for family planning**[1]	20.6*	25.7 (24.3-27.2)
**Adolescents 15-<20 years currently married**	5.2*	15.4*
**Age at first sex**		
**By age 15 years**	10.9*	14.1 (13.1-15.2)
**By age 20 years**	79.3*	90.0 (88.8-90.7)
**Age at first birth**		
**By age 15 years**	1.8*	2.6 (2.2-3.0)
**By age 20 years**	41.1*	58.9 (57.1-60.6)
**Parity**		
**0**	30.4*	22.6 (21.5-23.8)
**1**	17.9*	13.4 (12.6-14.3)
**2-3**	31.0*	26.8 (25.7-27.9)
**4-5**	14.5*	19.0 (17.9-20.0)
**6+**	6.3*	18.2 (17.1-19.4)
**Birth interval**		
**7-17 months**	2.0*	2.4 (2.0-2.9)
**18-23 months**	5.9*	9.7 (8.8-10.7)
**24-35 months**	25.3*	43.8 (42.0-45.6)
**36-47 months**	23.3*	21.8 (20.6-23.0)
**48+ months**	43.5*	22.3 (20.8-24.0)
**Anemia**		
**Severe**	3.9*	2.7 (2.1-3.3)

Laiser Y, *et al. BMJ Glob Health* 2026; 10:e017484. doi: 10.1136/bmjgh-2024-017484

Supplementary Table 3. Distribution of family planning and nutrition related measures among women of reproductive age across urban/rural status (prevalence, %, 95% confidence interval), Tanzania Demographic and Health Survey 2022.

	Urban	Rural
**Moderate**	22.7*	18.7 (17.2-20.3)
**Mild**	17.2*	19.0 (17.6-20.4)
**Not anemic**	56.3*	59.7 (57.5-61.8)
**BMI**		
**Underweight**	5.3*	6.3 (5.3-7.5)
**Normal**	49.5*	69.9 (68.0-71.8)
**Overweight**	25.5*	16.5 (15.1-18.0)
**Obese**	19.8*	7.3 (6.3-8.4)

[1]Unmet need for family planning examined among fecund, sexually active women.

*Estimates of 95% confidence interval not generated as subgroup sample size was insufficient for calculation.

Laiser Y, *et al. BMJ Glob Health* 2026; 10:e017484. doi: 10.1136/bmjgh-2024-017484

Supplementary Table 4. Prevalence of family planning indicators across measures of nutritional status among women of reproductive age (prevalence, %, 95% confidence interval), Tanzania Demographic and Health Survey 2022.

	Anemia				BMI			
	**Severe**	**Moderate**	**Mild**	**Not anemic**	**Underweight**	**Normal**	**Overweight**	**Obese**
**Current contraceptive method**								
**Not using**	77.0*	78.0*	71.3 (68.0-74.4)	64.9 (63.0-66.7)	72.5*	72.1 (70.2-74.0)	63.2 (60.0-66.3)	59.9*
**Modern methods**								
**Female sterilization**	3.6*	2.1*	1.9 (1.1-3.2)	2.1 (1.7-2.7)	0.6*	1.4 (1.1-1.8)	3.8 (2.8-5.3)	3.9*
**Male sterilization**	0.0*	0.0*	0.0 (0.0-0.0)	0.0 (0.0-0.2)	0.0*	0.0 (0.0-0.2)	0.1 (0.0-0.6)	0.0*
**Implants/norplant**	5.0*	6.4*	9.3 (7.4-11.6)	13.9 (12.6-15.2)	10.7*	11.1 (9.8-12.4)	11.8 (9.8-14.2)	12.3*
**Pill**	1.7*	1.4*	2.2 (1.3-3.6)	2.6 (2.1-3.3)	2.1*	2.1 (1.6-2.8)	2.8 (1.9-4.0)	2.3*
**Male condom**	2.1*	1.7*	1.9 (1.2-3.1)	1.9 (1.4-2.4)	1.5*	1.6 (1.2-2.1)	2.4 (1.5-3.6)	2.6*
**Periodic abstinence**	4.4*	3.7*	4.5 (3.3-6.2)	2.8 (2.2-3.5)	2.3*	2.7 (2.2-3.3)	3.3 (2.4-4.6)	7.5*
**Injections**	3.1*	4.2*	5.6 (4.4-7.1)	8.9 (7.9-10.1)	7.8*	6.6 (5.7-7.6)	9.1 (7.4-11.1)	6.9*
**IUD**	0.0*	0.3*	0.6 (0.3-1.2)	0.6 (0.3-1.0)	0.4*	0.3 (0.2-0.6)	0.7 (0.4-1.3)	1.4*
**Emergency contraception**	0.0*	0.1*	0.0 (0.0-0.0)	0.0 (0.0-0.0)	0.2*	0.0 (0.0-0.1)	0.0 (0.0-0.0)	0.0*
**Female condom**	0.0*	0.0*	0.0 (0.0-0.0)	0.0 (0.0-0.3)	0.0*	0.0 (0.0-0.0)	0.0 (0.0-0.0)	0.2*
**Traditional methods**								
**Withdrawal**	1.9*	1.7*	1.8 (0.1-2.9)	1.5 (1.0-2.0)	1.3*	1.2 (0.9-1.6)	2.2 (1.4-3.5)	2.6*
**Other traditional**	1.2*	0.3*	0.4 (0.2-1.1)	0.2 (0.0-0.4)	0.4*	0.3 (0.2-0.6)	0.3 (0.1-0.7)	0.0*
**Lactational amenorrhea**	0.0*	0.0*	0.5 (0.2-1.0)	0.6 (0.4-1.0)	0.2*	0.5 (0.3-0.8)	0.4 (0.2-0.9)	0.4*
**Standard days method**	0.0*	0.0*	0.0 (0.0-0.0)	0.0 (0.0-0.0)	0.0*	0.0 (0.0-0.0)	0.0 (0.0-0.0)	0.0*
**Age at first sex**								
**By age 15 years**	11.1*	10.4*	11.5 (9.5-14.0)	14.9 (13.2-16.7)	14.2*	12.7 (11.5-14.1)	13.7 (11.2-16.6)	14.8*
**By age 20 years**	84.7*	85.5*	85.4 (82.3-88.0)	86.8 (85.3-88.1)	79.9*	88.6 (87.3-89.8)	85.2 (82.6-87.4)	78.6*
**Age at first birth**								
**By age 15 years**	2.0*	1.9*	2.2 (1.5-3.3)	2.7 (2.1-3.4)	4.6*	1.9 (1.4-2.4)	3.2 (2.3-4.5)	3.1*
**By age 20 years**	48.3*	49.1*	50.6 (46.9-54.3)	55.2 (53.0-57.4)	41.7*	56.4 (54.2-58.5)	50.5 (47.4-53.6)	46.7*
**Parity**								
**0**	24.2*	29.9*	30.0 (27.2-33.0)	23.6 (22.0-25.2)	26.5*	30.4 (28.8-32.1)	19.8 (17.4-22.4)	12.5*
**1**	18.1*	15.5*	15.3 (13.0-18.0)	15.2 (14.0-16.6)	17.6*	15.1 (13.9-16.4)	15.9 (13.7-18.4)	15.1*
**2-3**	28.4*	26.1*	26.1 (23.4-29.0)	28.8 (27.0-30.6)	25.1*	25.7 (24.0-27.4)	29.7 (27.1-32.4)	37.7*
**4-5**	20.6*	15.6*	17.1 (14.8-19.7)	17.7 (16.3-19.2)	19.7*	15.2 (13.9-16.6)	19.6 (17.3-22.2)	23.0*

Laiser Y, *et al. BMJ Glob Health* 2026; 10:e017484. doi: 10.1136/bmjgh-2024-017484

Supplementary Table 4. Prevalence of family planning indicators across measures of nutritional status among women of reproductive age (prevalence, %, 95% confidence interval), Tanzania Demographic and Health Survey 2022.

	Anemia				BMI			
	Severe	Moderate	Mild	Not anemic	Underweight	Normal	Overweight	Obese
**6+**	8.7*	12.9*	11.4 (9.5-13.7)	14.7 (13.3-16.2)	11.1*	13.6 (12.4-15.0)	15.0 (12.7-17.6)	11.7*
**Birth interval**								
**7-17 months**	0.0*	2.0*	3.2 (2.0-5.2)	2.1 (1.4-3.2)	4.7*	2.0 (1.4-2.9)	2.2 (1.2-3.9)	2.2*
**18-23 months**	6.2*	9.4*	9.4 (7.3-12.1)	7.2 (6.0-8.5)	7.7*	8.7 (7.4-10.1)	7.6 (5.8-9.8)	5.5*
**24-35 months**	42.1*	38.8*	33.5 (29.4-37.9)	39.4 (36.9-41.9)	45.4*	43.0 (40.4-45.7)	32.7 (29.4-36.2)	24.5*
**36-47 months**	26.1*	21.3*	23.7 (20.3-27.4)	22.6 (20.6-24.7)	19.9*	22.8 (20.7-25.1)	23.6 (20.7-26.9)	21.3*
**48+ months**	25.6*	28.5*	30.2 (26.3-34.5)	28.8 (26.6-31.1)	22.4*	23.5 (21.3-25.9)	33.9 (30.4-37.6)	46.5*

*Estimates of 95% confidence interval not generated as subgroup sample size was insufficient for calculation.

Supplementary Table 5. Risk of anemia, underweight and overweight/obesity associated with family planning and related measures among women of reproductive age, Tanzania Demographic and Health Survey 2022.

	Anemia		Underweight		Overweight/obesity	
	**Risk ratio (95% CI)**	***P***	**Risk ratio (95% CI)**	***P***	**Risk ratio (95% CI)**	***P***
**Current contraceptive method**						
**Not using**	1.00		1.00		1.00	
**Modern methods**						
**Female sterilization**	0.96 (0.77-1.21)	0.751	0.45 (0.11-1.81)	0.258	1.22 (1.04-1.43)	0.015
**Male sterilization**	-	-	-	-	1.86 (0.89-3.85)	0.097
**Implants/norplant**	0.63 (0.54-0.72)	<0.001	0.80 (0.51-1.25)	0.322	1.04 (0.92-1.17)	0.536
**Pill**	0.72 (0.55-0.95)	0.018	0.77 (0.41-1.47)	0.434	0.99 (0.77-1.27)	0.928
**Male condom**	0.91 (0.71-1.18)	0.488	0.90 (0.43-1.90)	0.789	1.05 (0.83-1.33)	0.692
**Periodic abstinence**	1.12 (0.93-1.35)	0.238	0.64 (0.29-1.44)	0.283	0.93 (0.80-1.07)	0.309
**Injections**	0.62 (0.52-0.73)	<0.001	0.97 (0.65-1.45)	0.901	1.10 (0.95-1.28)	0.213
**IUD**	0.81 (0.48-1.37)	0.438	1.11 (0.28-4.39)	0.878	1.20 (0.92-1.57)	0.174
**Emergency contraception**	2.08 (1.87-2.32)	<0.001	4.76 (1.90-11.96)	0.001	0.47 (0.05-4.16)	0.500
**Female condom**	-	-	-	-	2.41 (2.11-2.76)	<0.001
**Traditional methods**						
**Withdrawal**	1.04 (0.79-1.35)	0.794	0.95 (0.38-2.37)	0.917	1.23 (0.96-1.57)	0.104
**Other traditional**	1.41 (0.96-2.09)	0.083	1.07 (0.25-4.66)	0.929	0.58 (0.26-1.30)	0.185
**Lactational amenorrhea**	0.45 (0.21-0.95)	0.037	0.29 (0.04-2.06)	0.217	0.95 (0.61-1.48)	0.808
**Standard days method**	2.13 (1.86-2.43)	<0.001	-	-	-	-
**Age at first sex**						
**By age 15 years**	0.80 (0.70-0.90)	<0.001	1.01 (0.73-1.39)	0.942	1.17 (1.05-1.30)	0.004
**By age 20 years**	0.93 (0.83-1.03)	0.167	0.59 (0.44-0.80)	0.001	1.05 (0.94-1.18)	0.342
**Age at first birth**						
**By age 15 years**	0.83 (0.65-1.08)	0.165	1.93 (1.09-3.43)	0.025	1.36 (1.13-1.63)	0.001
**By age 20 years**	0.87 (0.80-0.94)	0.001	-	-	1.07 (0.98-1.16)	0.127
**Parity**						
**0**	1.00		1.00		1.00	
**1**	0.87 (0.77-0.97)	0.013	0.68 (0.49-0.97)	0.031	1.11 (0.95-1.28)	0.187
**2-3**	0.79 (0.71-0.88)	<0.001	0.49 (0.34-0.69)	<0.001	1.11 (0.97-1.27)	0.137
**4-5**	0.79 (0.70-0.89)	<0.001	0.59 (0.41-0.85)	0.004	1.12 (0.94-1.34)	0.214
**6+**	0.71 (0.61-0.82)	<0.001	0.38 (0.23-0.63)	<0.001	1.01 (0.81-1.26)	0.909

Laiser Y, *et al. BMJ Glob Health* 2026; 10:e017484. doi: 10.1136/bmjgh-2024-017484

Supplementary Table 5. Risk of anemia, underweight and overweight/obesity associated with family planning and related measures among women of reproductive age, Tanzania Demographic and Health Survey 2022.

	**Anemia**		**Underweight**		**Overweight/obesity**	
	**Risk ratio (95% CI)**	***P***	**Risk ratio (95% CI)**	***P***	**Risk ratio (95% CI)**	***P***
**Birth interval**						
**7-17 months**	1.00		1.00		1.00	-
**18-23 months**	1.06 (0.73-1.54)	0.745	0.39 (0.18-0.81)	0.013	0.91 (0.67-1.23)	0.527
**24-35 months**	0.89 (0.63-1.24)	0.485	0.47 (0.24-0.95)	0.035	0.77 (0.59-1.01)	0.056
**36-47 months**	0.93 (0.66-1.31)	0.682	0.41 (0.20-0.83)	0.013	0.86 (0.65-1.14)	0.303
**48+ months**	0.94 (0.67-1.32)	0.716	0.47 (0.21-1.04)	0.062	0.98 (0.74-1.29)	0.884

Estimates based on survey-weighted Poisson regression models, adjusted for age category, wealth quintile, education status and rural/urban status.

95% CI: 95% confidence interval. -: no or insufficient observations.

Laiser Y, *et al. BMJ Glob Health* 2026; 10:e017484. doi: 10.1136/bmjgh-2024-017484

Supplementary Table 6. Summary of reviewed policy and programme documents covering family planning and nutrition in Tanzania.

Document & year	Goals and objectives, relevant target population	Target population	Relevant overarching targets	Key activities	Platform of delivery	Process of development	Other domains integrated (or specific domains covered for general documents)
**Documents on family planning**							
**National documents**							
National Family Planning Guideline and Standards, 2013	Provide explicit directives on:<br>1. Operational rules, regulations and administrative norms governing FP services; and,<br>2. Minimum acceptable levels of performance and expectations for quality service delivery and programme implementation in Tanzania.	Managers, supervisors and service providers offering FP services in all governmental, non-government and faith-based organizations and private sector at all levels of the health system.	Reducing total fertility rate from 5.4 in 2010 to 5.0 by 2015ss.	Training implementers to ensure that they remain accountable to common and uniform guidelines and standards to ensure quality FP provision in Tanzania	All health facilities implementing FP services including home-based care, dispensaries, health centers, hospitals, non-government and faith-based organizations pharmacies, and community facilities such as schools, health posts and other community settings	1. Stakeholder consultations with representatives from the government, civil society organizations, and development partners<br>2. Situation analysis of the status of FP services in the country	1. National Reproductive and Child Health Strategy and the Health Sector Strategic Plan<br>2. National HIV/AIDS Control Programme
National Family Planning Costed Implementation Programme, 2010	**Goal:** Increase the contraceptive prevalence rate (CPR) among women of reproductive age from 28% to 60%by 2015<br><br>**Objectives**:<br>1.Expand access to a range of safe, effective, and affordable contraceptive methods.<br>2. Build provider capacity for safe and effective FP service delivery.<br>3. Strengthen service delivery systems and increase sustainable FP options.<br>4. Reinvigorate advocacy to promote FP as a key investment in public health and well-being.<br>5. Strengthen health system management and	Women of reproductive age and adolescents, and special focus on poor and marginalized communities	1. Increase CPR to 60% by 2015<br>2. Reduce unmet needs for FP to 22% in 2015<br>3. Improve skilled attendance at birth to 50% in 2015	1. Increasing access to FP services<br>2. Strengthening of health system<br>3. Integration of FP into other services<br>4. Training of healthcare providers<br>5. Increasing outreach services<br>6. Improving quality of FP services<br>7. Increasing demand for FP services	Service delivery points such as health facilities, mobile clinics, and Community-based and outreach services	Not specified	Not specified

Supplementary Table 6. Summary of reviewed policy and programme documents covering family planning and nutrition in Tanzania.

Document & year	Goals and objectives, relevant target population	Target population	Relevant overarching targets	Key activities	Platform of delivery	Process of development	Other domains integrated (or specific domains covered for general documents)
	M&E of the national FP programme.						
National Family Planning Costed Implementation Programme, 2019	Enable women, youth, and couples to achieve their desired fertility intentions through access to high-quality and respectful services as well as appropriate, evidence-based information	Women of reproductive age with their partners (couples) and youth	Increase the modern CPR to 47% among married women and 40% among all women by 2023.	1.Increase uptake of postpartum FP.<br>2. Address social norms hindering contraceptive use.<br>3. Reduce stockouts at facilities to ensure a full range of contraceptive methods.<br>4. Improve access to age-appropriate contraceptive information and services for young people (10–24 years).	Health delivery points such as health facilities, mobile clinics, community-based distribution and outreach services.	1. Situation analysis results	None
National Road Map Strategic Plan to Improve Reproductive, Maternal, Newborn, Child and Adolescent Health in Tanzania - One Plan II, 2016	Improve reproductive, maternal, newborn, child & adolescent health in line with the National Developmental Vision 2025.	All citizens of Tanzania	1. Increase modern CPR from 27% to 45% by 2020<br>2. Increase new modern FP clients from 2.6 million in 2015 to 4.2 million in 2020<br>3. Expand outreach-delivered FP services from 15.2% to 30% by 2020<br>4. Increase couple years of protection by all modern methods from 4.3 million to 6.4 million by 2020<br>5. Increase male HIV testing during provider-initiated testing and counseling interventions from 8% to 30% by 2020	Enhance contraceptive coverage by training providers, conducting outreach, partnering with the private sector, updating FP curricula in health institutions, and ensuring the procurement, distribution, and quantification of FP commodities.	Health delivery points such as health facilities and community-based distribution.	1. Stock of the lessons learnt from the millennium development goals and new evidence on effective investments and action.<br>2. Review of national health policy and strategies.<br>3. Lessons learnt during implementation of One Plan I	1.Prevention of Mother to Child Transmission of HIV<br>2.Cancer and health services to elderly<br>3. Gender Based Violence and Violence against Children
National Family Planning Procedure Manual, 2011	Overview the scope of family planning service delivery, covering steps for provision, elements of	FP stakeholders, managers, supervisors,	Not specified	FP service delivery, infection prevention, education and	Health care facilities	Review of previous versions of procedure manuals	1. Maternal, Newborn and Child Health Care

Supplementary Table 6. Summary of reviewed policy and programme documents covering family planning and nutrition in Tanzania.

Document & year	Goals and objectives, relevant target population	Target population	Relevant overarching targets	Key activities	Platform of delivery	Process of development	Other domains integrated (or specific domains covered for general documents)
	quality care (client rights and provider needs), key components of quality FP services, roles of trained healthcare providers, clinic organization, and logistics management.	trainers, and service providers		counseling strategies for FP and reproductive health services, provider-initiated testing and counseling, client screening for FP methods, and descriptions of short-acting, long-acting, and permanent FP methods.			
Delivering Inclusive Family Planning Services to Tanzanian Communities (Report on scaling up family planning programme by EngenderHealth), 2022	**Goal**: Enhance the capacity of the national health system to deliver inclusive and comprehensive sexual reproductive health services to 2.3 million Tanzanians<br>**Objectives:**<br>1. Increase utilization of integrated FP services, including postpartum FP, and screening for HIV, STIs, and cervical cancer.<br>2. Strengthen comprehensive post-abortion care.<br>3. Improve response to sexual and gender-based violence cases at community, facility, and health system levels.<br>4. Strengthen health systems.	Population covered by 545 health facilities in eight regions across mainland Tanzania and five regions of Zanzibar	Not specified	Outreach services, routine services, postpartum FP, training of healthcare providers, service quality audits, technical assistance to regional and council health management teams, and data quality and contraceptive commodity availability assessment	Health delivery points such as outreach and healthcare facilities	Not specified	1. Maternal and obstetric care,<br>2. Screening and follow-up services for GBV<br>3. Routine screening and treatment referrals for cervical cancer and HIV
**International documents**							

Supplementary Table 6. Summary of reviewed policy and programme documents covering family planning and nutrition in Tanzania.

Document & year	Goals and objectives, relevant target population	Target population	Relevant overarching targets	Key activities	Platform of delivery	Process of development	Other domains integrated (or specific domains covered for general documents)
FP2020 Partnership in Progress 2013-2014,	Enable an additional 120 million women and girls to use modern methods of contraception by the year 2020.	Women and girls of reproductive age	To increase the CPR from 27% to 60% by 2015	UNFPA supports government efforts by advocating for family planning, contraceptive procurement and supply, mobile outreaches and awareness campaigns.	Health delivery points such as clinics and pharmacies	Core indicators derived from the London Summit commitments were analyzed annually leading to the formation of this report.	1. Maternal healthcare programme and other health services
**Documents covering nutrition**							
**National documents**							
National Nutrition Strategy, 2011	All Tanzanians to attain adequate nutritional status, which is an essential requirement for a healthy and productive nation.	Children of school age, the youth, and the elderly, persons infected or affected by HIV and AIDS, and vulnerable children who have little or no access to health services and affected by emergencies and disasters	1. Reduce underweight in children (0–59 months) from 16% to 11%, stunting from 42% to 27%, vitamin A deficiency in children (6–59 months) from 24% to <15%, anemia in pregnant women from 48.4% to 35%, and anemia in children (6–59 months) from 71.8% to 55%.<br>2. Increase exclusive breastfeeding in infants (<6 months) from 50% (2010) to 60%<br>3. Maintain wasting in children (0–59 months) below 5%, thinness in women of reproductive age below the 2005 level of 10%, and iodine deficiency in children (6–12 years) below 50%	1. Expand access to quality nutrition services and build capacity at community, district, and regional levels.<br>2. Enforce nutrition-related legislation, integrate nutrition into national policies, and secure sustainable financing.<br>3. Strengthen monitoring, evaluation, research frameworks, and partnerships to improve nutrition interventions.	1. Public health facilities<br>2. Higher learning and training institutions<br>3. Professional nutrition bodies<br>4. Private health facilities<br>5. Development Partners<br>6. Civil society<br>7. Media<br>8. Community	A participatory process involving nutrition stakeholders at various levels	1. Maternal and child health

Supplementary Table 6. Summary of reviewed policy and programme documents covering family planning and nutrition in Tanzania.

Document & year	Goals and objectives, relevant target population	Target population	Relevant overarching targets	Key activities	Platform of delivery	Process of development	Other domains integrated (or specific domains covered for general documents)
Tanzania Agriculture and Food Security Investment Plan (TAFSIP), 2011	Contribute to the national economic growth, household income and food security in line with national and sectoral development aspirations growth, household income and food security in line with national and sectoral development aspirations	Agriculture and food sector stakeholders	1. At least 6% per annum growth of agricultural sector output<br>2. Sustainable and responsible natural resource management<br>3. Improve agricultural productivity<br>4. Increase calorie availability per rural household<br>5. Reduce prevalence of micronutrient deficiencies<br>6. Improved food quality, diversity, and reduced prevalence of malnutrition	1. Enhance government-donor commitment with agreed criteria/indicators for objective performance tracking.<br>2. Establish dialogue platforms based on mutual consent, shared values, and trust for review processes.<br>3. Strengthen M&E systems to track indicators and generate performance and impact data.	Not specified	Not specified	Maternal, neonatal and child health
Infant and Young Child Feeding National Guidelines, 2013	Improve the nutritional status, growth and development, health and survival of infants and young children through optimal feeding practices.	Health care providers and professionals, and institutions such as health facilities	Not specified	Not specified	The guidelines are intended to be used as an operational tool for the healthcare providers at different levels.	The National Guidelines on IYCF are informed by international instruments, national policies, strategies, and related guidelines, summarizing recommendations for infant and young child feeding at various ages.	Maternal, neonatal and child health
Lishe Endelevu — Sustainable nutrition (2018-2023), 2019	Strengthen local government capacity, coordination with civil society and the private sector, access to quality nutrition services, household production of nutrient-rich foods, and equitable control over	Women of reproductive age, children under 5yrs, adolescent girls 15-19yrs	To reach over 1.6 million women of reproductive age, 1.1 million children under age 5, and 364,000 adolescent girls 15-19 years of age in Dodoma, Iringa, Morogoro and Rukwa regions.	1.Train LGAs and health staff to align activities with NMNAP and build health workers' capacity for improved nutrition services.	Through training institutions and village demonstration plots for capacity building but also through healthcare delivery points such as community outreach	Not specified	Water, sanitation and hygiene (WASH)

Supplementary Table 6. Summary of reviewed policy and programme documents covering family planning and nutrition in Tanzania.

Document & year	Goals and objectives, relevant target population	Target population	Relevant overarching targets	Key activities	Platform of delivery	Process of development	Other domains integrated (or specific domains covered for general documents)
	resources for dietary diversity.			2. Promote women's leadership in shaping local nutrition agendas and engage nutrition stakeholders from civil society and the private sector.<br>3. Support CHWs/Volunteers and model farmers to establish demonstration plots for improved agriculture, livestock, aquaculture, and nutrition behaviors.	services as well as healthcare facilities both public and private		
Tanzania Food and Nutrition Centre Strategic Plan (2005/2006 – 2009/2010), 2006	Improve the performance and service delivery of the Centre and ultimately contribute towards improvement of the nutrition situation in the country.	All citizens of Tanzania	1. Supporting 50 councils in establishing and operating Community Based Nutrition Rehabilitation<br>2. Reduction in the problem of low birth weight through improved maternal care in 50 districts<br>3. Knowledge and skills on Growth Monitoring and Promotion imparted to health workers providing Reproductive and Child Health Services in all districts<br>4. Policy guidelines on Infant and Young Child Nutrition developed and disseminated by 2010.<br>5. Consumption of vitamins and mineral rich	Conducting activities for promotion of nutrition services such as Community Based Nutrition Rehabilitation, strengthening partnerships with both public and private sector especially in policy making, dissemination and advocacy. Other activities include capacity building and maternal care services such as micronutrient supplementation and dietary improvement.	Service to be delivered through Community Based Nutrition Rehabilitation Centers and healthcare delivery points such as public and private health care facilities	1. The working group within TFNC produced a draft copy of strategic plan which was discussed by all TFNC programme officers through a one-day internal seminar presentation.<br>2. The outcome was presented to the TFNC Board for their inputs.<br>3. The draft, which included the inputs from the Board, was distributed to other key stakeholders for further inputs.	Not specified

Supplementary Table 6. Summary of reviewed policy and programme documents covering family planning and nutrition in Tanzania.

Document & year	Goals and objectives, relevant target population	Target population	Relevant overarching targets	Key activities	Platform of delivery	Process of development	Other domains integrated (or specific domains covered for general documents)
			foods promoted particularly to vulnerable groups.				
National Guidelines for Nutrition Care and Support of People with HIV, 2016	1.Harmonized guidance for nutrition care and support for people with HIV across age groups, sectors, and communities.<br>2. Framework for planning, implementation, and monitoring nutrition care for people with HIV.<br>3. Enhance front-line providers' knowledge and skills for quality HIV-related nutrition care.<br>4. Guidance on continuous care for malnourished people with HIV at health and community levels.	People living with HIV	Not specified	1. Nutrition assessment<br>2. Nutrition education and counselling<br>3. Therapeutic and/or supplementary feeding<br>4. Referral to follow-up care and other needed services such as food security and social safety net programmes	Services can be provided through health care facilities and community mobilization in terms of community support groups, community outreaches as well as home-based care.	The National AIDS Control Programme provided the policy and technical context. WHO and UNICEF provided a thorough technical review of the updated guidelines.	WASH
National Multisectoral Nutrition Action Plan (2021/22 - 2025/26), 2021	Provide directions to the nutrition stakeholders at all levels on the implementation of nutrition interventions, which address the triple burden of malnutrition in the country for the next five years from 2021	All citizens of Tanzania	1. Reduce stunting in children (0-59 months) to 24%, low birthweight to <5%, and anemia in non-pregnant women (15-49 years) to 23%, while maintaining global acute malnutrition in children under 5 at <5% by 2025/26.<br>2. Increase exclusive breastfeeding in children (0-5 months) to 70%, minimum acceptable diet in children (6-23 months) to 50%, Vitamin A supplementation in children (6-59 months) to 90%, and SAM treatment coverage for children under five to 75% by 2025/26.	1. Promotion of continued and consistent use of iron and folic acid<br>2. Promotion of diversified diet<br>3. Nutrition assessment and counseling<br>4. Promotion of locally available foods and fortified foods<br>5. Vitamin A supplementation for under fives<br>6. Management of acute malnutrition<br>7. Provision of school meals with diversified and fortified foods	Services to be provided in schools and other health delivery points such as healthcare facilities and community outreach	Not specified	Not specified

Supplementary Table 6. Summary of reviewed policy and programme documents covering family planning and nutrition in Tanzania.

Document & year	Goals and objectives, relevant target population	Target population	Relevant overarching targets	Key activities	Platform of delivery	Process of development	Other domains integrated (or specific domains covered for general documents)
Tanzania Food and Nutrition Centre Strategic Plan (2016/17 - 2020/21), 2016	1. Coordination of nutrition and nutrition related activities Strengthened<br>2. Research and training on subjects related to food and nutrition improved<br>3. Food and nutrition programmes for the benefit of the public strengthened<br>4. National food and nutrition information and data improved<br>5. Planning, budgeting, financial controls and soliciting of funds strengthened<br>6. Staff welfare and capacity enhanced	All citizens of Tanzania	1. Develop new guidelines, strategies, protocols, and regulations by June 2018, and operationalize the national food and nutrition information system by June 2018.<br>2. Conduct research, monitoring, and evaluation exercises, and provide training on food and nutrition, while reviewing or developing plans and curricula by June 2017–2021.	1.Develop and implement programmes to address HIV/AIDS, and noncommunicable diseases, while providing supportive services to reduce infection.<br>2. Strengthen anti-corruption measures, review outdated policies, and develop new guidelines, strategies, and regulations.<br>3. Establish zonal offices, conduct monitoring and evaluation on nutrition activities, and build capacity for research and training in food and nutrition.	Not specified	The process of reviewing the Plan was participatory, heads of departments from within and other from independent institutions were involved at the initial stage. Before finalization of the document, other staffs were also involved.	1. Sexual and reproductive health<br>2. Maternal, neonatal and child health
**Regional documents**							
Southern African Development Community Food and Nutrition Security Strategy (2015 – 2025), 2014	Significantly reduce food and nutrition insecurity in the Region by 2025.	Implementers from SADC member states	1. Reduce stunting prevalence (height-for-age) to below 30% by 2025.<br>2. Align wasting (weight-for-height), underweight (weight-for-age), anemia, low birth weight, and exclusive breastfeeding prevalence with global targets.	1. Improve food productivity, access to land and water, and reduce post-harvest losses.<br>2. Enhance climate change adaptation, access to credit and markets, and support income generation and social protection.<br>3. Promote access to labor-saving	Not specified	Not specified	Not specified

Supplementary Table 6. Summary of reviewed policy and programme documents covering family planning and nutrition in Tanzania.

Document & year	Goals and objectives, relevant target population	Target population	Relevant overarching targets	Key activities	Platform of delivery	Process of development	Other domains integrated (or specific domains covered for general documents)
				technologies, improved infant and young child nutrition, and reduce micronutrient deficiencies for sustainable food availability, access, and utilization.			
**Documents including components of family planning and nutrition**							
**National documents**							
National Accelerated Action and Investment Agenda for Adolescent Health and Wellbeing (2021/22-2024/25), 2021	Accelerate the improvement of adolescent health and wellbeing to support the growth and development of healthy, educated and empowered adolescents as they transition into adulthood.	Adolescents	1.Reduce new infections and unwanted pregnancies (from 27% to 5% by 2021).<br>2. Achieve 70% of schools with approved reproductive health curricula and 80% of health facilities providing adolescent-friendly services by 2020.<br>3. Increase modern contraceptive use among sexually active women aged 10–19 (40% for all, 47% for married) and condom use among 15-19-year-olds (85%) by 2022, while reducing HIV incidence by 50% in adolescents by 2022.	Activities span from prevention activities such as youth friendly community-based testing to capacity building activities for healthcare workers, TOTs and community leaders. Other activities include school activities such as establishment of Child Protection Desks, regular school check-ins by social workers, school garden activities and social and behavior change activities.	Health delivery points such as outreach and healthcare facilities as well as other platforms such as schools and media	This document was developed building on several other relevant policy documents such as the National Health Policy, National Policy on HIV/AIDS, Health Sector Strategic Plan IV, National Multisectoral Nutrition Action Plan II, One Plan II and National Family Planning Costed Implementation Plan II.	Not specified
Policy Guidelines on School Health Services in Tanzania, 2018	Revise and update the National School Health Programme to fulfil the Sustainable Development Goals related to child health, gender, equality, universal education	School children	1. Reduce hunger and improve the nutritional status of school children.<br>2. Increase school enrolment, attendance, retention, and completion rates, especially for	Not specified	Schools	Stakeholders contributed to the development of the Guideline through brainstorming, analyzing the situation, and recommending key	Adolescent health

Supplementary Table 6. Summary of reviewed policy and programme documents covering family planning and nutrition in Tanzania.

Document & year	Goals and objectives, relevant target population	Target population	Relevant overarching targets	Key activities	Platform of delivery	Process of development	Other domains integrated (or specific domains covered for general documents)
			children from poor communities.<br>3. Enhance learners' comprehension, learning abilities, and environmental sustainability.			issues. They also provided support by offering constructive critiques and feedback on earlier drafts.	
Tanzania Primary Health Services Development Programme (2007 – 2017), 2007	Contribute to the national economic growth, household income and food security in line with national and sectoral development aspirations growth, household income and food security in line with national and sectoral development aspirations	Rural areas and those in the vulnerable population	1.Increase the availability of basic health services in all facilities and establish a health facility in every village by 2012.<br>2. Ensure the availability of essential medicines, medical supplies, and equipment in public primary health facilities and enhance capacity for nutrition at district and community levels.<br>3. Improve infrastructure by acquiring vehicles and ambulances, procuring radio systems for districts, and rehabilitating/upgrading health facilities, including strengthening health centers with necessary equipment.	1.Recruit and deploy skilled personnel, ensure availability and management of drugs, supplies, and equipment, and rehabilitate and construct health facilities.<br>2. Provide mobile clinics for outreach services, allocate adequate budgets for medicines, and improve delivery systems for medical supplies in primary health facilities.<br>3. Ensure availability of guidelines in primary health facilities and establish planning and standardized stock-control systems.	Health delivery points such as healthcare facilities and mobile clinics	Situational analysis of various aspects of primary health care by using data from various studies and surveys such as Tanzania Service Provision Assessment Survey (2006) and Population Census (2002)	1. Family planning<br>2. Antenatal care
The National Strategy for Growth and Reduction of Poverty II	1. Bring health care services closer to the people, at a distance of not more than 5 kilometers<br>2. Improve referral system at all levels and availability	All citizens of Tanzania	1.Achieve agricultural growth (2.7% to 6%), livestock growth (2.3% to 4.5%), crops growth (3.4% to 6.4%), and fisheries	1.Promote new farming practices for crops, livestock, and fish with high nutritional content, and increase fish	Not specified	1. Preparatory stage<br>2. The Assessment Stage<br>3. Drafting and Dialogue Phase	1. Nutrition<br>2. Maternal, newborn and child health

Supplementary Table 6. Summary of reviewed policy and programme documents covering family planning and nutrition in Tanzania.

Document & year	Goals and objectives, relevant target population	Target population	Relevant overarching targets	Key activities	Platform of delivery	Process of development	Other domains integrated (or specific domains covered for general documents)
(MKUKUTA II), 2010	of health workers including doctors and nurses		growth (2.7% to 5.3%) by 2015.<br>2. Promote nutrition for infants, young children, and mothers, ensure food security, and introduce climate-resilient crop and livestock varieties.<br>3. Reduce under-five underweight (21% to 14%), stunting (35% to 22%), and anemia prevalence (women: 48.4% to 35%, children: 71.8% to 55%), while increasing exclusive breastfeeding (50% to 60%) by 2015.	production through aquaculture.<br>2.Improve sustainable fisheries management, promote exclusive breastfeeding, and support research on climate-resilient agricultural varieties.<br>3. Enhance food storage and preservation technologies and ensure universal Vitamin A coverage for under-five children and postpartum women.		4. Stakeholder Consultations	
Human Resource for Health Strategic Plan, 2008	1.Improve HR planning and policy development, strengthen leadership and stewardship, and enhance education and training for the health workforce.<br>2. Enhance workforce management, utilization, and foster partnerships in Human Resources for Health.<br>3. Strengthen HRH research and development and promote adequate financing for the HRH strategic plan.	All citizens of Tanzania	1.Enhance leadership and stewardship capacity in the public and private health sectors and strengthen zonal training centers for quality healthcare delivery and training.<br>2. Improve quality assurance systems in all health facilities and strengthen human resource performance management and reward systems.<br>3. Foster partnerships among Human Resource for Health stakeholders and promote HRH research for effective planning, decision-making, and advocacy.	1.Build capacity in information management, policy analysis, planning, monitoring, and evaluation across all levels.<br>2. Strengthen policy analysis and interpretation and establish leadership and management programmes in the health sector.<br>3. Develop a training master plan and scale up health worker enrollment and training.	Healthcare Facilities, training Institutions and centers for People with disabilities.	1. Consultations<br>2. Working sessions and discussions<br>3. Document review<br>4. Use of study findings and presentations	Not specified

Laiser Y, *et al. BMJ Glob Health* 2026; 10:e017484. doi: 10.1136/bmjgh-2024-017484

Supplementary Table 6. Summary of reviewed policy and programme documents covering family planning and nutrition in Tanzania.

Document & year	Goals and objectives, relevant target population	Target population	Relevant overarching targets	Key activities	Platform of delivery	Process of development	Other domains integrated (or specific domains covered for general documents)
The National Health Policy, 2017	Reach all households with essential health services attaining the needs of the population, adhering to objective quality standards and applying evidence-informed interventions through resilient systems for health.	All citizens of Tanzania	Not specified	1.Strengthen governing principles, regulations, and guidelines for health and nutrition services, promoting community engagement and stakeholder linkages.<br>2.Enhance mechanisms for nutrient supplementation, fortification, and dietary interventions to address micronutrient deficiencies.<br>3. Promote appropriate maternal and child feeding practices, raise awareness on lifestyle diseases, and advocate for food security and local food research.	Healthcare facilities	The policy was developed by revising the National Health Policy 2007 and updating it with new information and government commitments	1. Nutrition<br>2. Reproductive, Maternal, Newborn, Child and Adolescent Health
Tanzania-Health-Sector-Strategic-Plan-V (HSSP V), 2021	1.Ensure accessibility and availability of basic health services for all, and control communicable and non-communicable diseases.<br>2. Increase public awareness on preventable diseases and promote individual responsibility for health care.	All citizens of Tanzania	1.Achieve 65% early initiation of breastfeeding and reduce stunting in children under 5 by 20%, ensuring all regions have stunting rates below 25%.<br>2. Decrease anemia prevalence by 25% in women (15-49 years), adolescents (15-19 years), and under-fives.	Not specified	All health stakeholders	1. Consultation with stakeholders<br>2. Roundtable with stakeholders<br>3. Stakeholder engagement	1. Family planning<br>2. Sexual and reproductive health<br>3. Maternal, neonatal and child health

**Supplementary Table 6. Summary of reviewed policy and programme documents covering family planning and nutrition in Tanzania.**							
**Document & year**	**Goals and objectives, relevant target population**	**Target population**	**Relevant overarching targets**	**Key activities**	**Platform of delivery**	**Process of development**	**Other domains integrated (or specific domains covered for general documents)**
	3. Strengthen partnerships across sectors, enhance health workforce training, and maintain health infrastructure and equipment.		3. Prevent an increase in overweight and obesity among adults (15-59 years) and ensure 80% of households have access to safe drinking water.				
National Management Guidelines for the Health Sector Response to and Prevention of Gender-Based Violence (GBV), 2011	Provide standards for the provision of high-quality and comprehensive medical services and procedures to GBV survivors, and encourage providers to identify and quickly mobilize the required resources, materials and essential medication for GBV, at health facilities	Healthcare providers, and health managers	Not specified	1.Integrate GBV services into existing health services and train healthcare providers to support victims effectively.<br>2. Provide family planning counseling and contraceptive methods to women of reproductive age who are GBV victims.<br>3. Offer emergency contraception to GBV survivors of child-bearing age.	Healthcare facilities, drop-in centers and safe houses	Not specified	1. Emergency Contraception<br>2. Mental Health
Strengthening Postpartum Family Planning (PPFP) and Maternal, Infant and Young Child Nutrition (MIYCN) Outcomes in Mara and Kagera, Tanzania A Multi-level Approach, 2019	Assess the impact of the multi-level facility and community intervention to integrate Maternal Infant Young Child Nutrition (MIYCN) and Postpartum Family Planning (PPFP) within existing health contacts in Mara and Kagera	Lactating mothers with infants less than 6 months of age	Not specified	1. Engage influential community members and provide LAM tracking and follow-up tools.<br>2.Develop job aids, self-monitoring tools, and songs to support LAM counseling by health workers and CHWs.<br>3.Conduct onsite training and	Health facilities	Not specified	1. Maternal, Infant and Young Child Nutrition<br>2. Family Planning

Supplementary Table 6. Summary of reviewed policy and programme documents covering family planning and nutrition in Tanzania.

Document & year	Goals and objectives, relevant target population	Target population	Relevant overarching targets	Key activities	Platform of delivery	Process of development	Other domains integrated (or specific domains covered for general documents)
				monthly supportive supervision for health workers and CHWs.			

Laiser Y, *et al. BMJ Glob Health* 2026; 10:e017484. doi: 10.1136/bmjgh-2024-017484
